# Peripheral blood biomarkers for predicting response to PD-1/PD-L1 inhibitors

**DOI:** 10.1186/s40364-026-00892-5

**Published:** 2026-01-14

**Authors:** Shuting Chen, Yingjie Hu, Hanxu Cheng, Shuaiqingying Guo, Yingyan Han, Kezhen Li

**Affiliations:** 1 Department of Gynecology and Obstetrics, Tongji Hospital, Tongji Medical College, Huazhong University of Science and Technology, Wuhan, China; 2 National Clinical Research Center for Obstetrics and Gynecology, Cancer Biology Research Center (Key Laboratory of the Ministry of Education), Tongji Hospital, Tongji Medical College, Huazhong University of Science and Technology, Wuhan, China

**Keywords:** Peripheral blood, Predictive biomarker, PD-1/PD-L1 inhibitors, Tumor-derived biomarker, Immune cell, Cytokines, Multi-parameter model

## Abstract

Though programmed cell death protein 1/programmed cell death ligand 1 (PD-1/PD-L1) inhibitors have revolutionized cancer treatment, predicting their therapeutic efficacy largely depends on biomarkers obtained through invasive procedures, which present challenges, particularly for continuous sampling. Compared to tumor-based biomarkers, peripheral blood biomarkers offer distinct advantages, including non-invasive accessibility and the potential for dynamic monitoring. Although numerous studies suggest that peripheral blood biomarkers hold significant value, the lack of consensus on indicators and standards underscores the necessity of integrating evidence to guide future research. This review synthesizes cutting-edge peripheral blood testing achievements for predicting PD-1/PD-L1 immunotherapy response, encompassing tumor-derived biomarkers (circulating tumor cell, circulating tumor DNA, blood tumor mutation burden, etc.), multidimensional immune profiling (lymphocyte count, neutrophil-to-lymphocyte ratio, T/B/NK/myeloid subsets, cytokines), and multi-parameter models. We collectively validate the reliability of peripheral blood biomarkers and identify high-potential candidates that could be further developed through computationally-driven frameworks and multi-center cohorts in the future.

## Background

In 2014, the U.S. Food and Drug Administration (FDA) granted accelerated approval to nivolumab and pembrolizumab for advanced melanoma treatment, establishing these agents as the first programmed death 1 (PD-1) inhibitors to receive regulatory authorization [1]. Following their initial approval, both therapeutic agents obtained subsequent indications for multiple malignancies [2], demonstrating their broad clinical applicability across diverse oncological contexts. Meanwhile, more PD-1/programmed cell death ligand 1 (PD-L1) inhibitors have been approved for clinical use. To date, the FDA has approved several biomarkers for predicting therapeutic efficacy, including tissue PD-L1 expression, tumor mutation burden (TMB), and microsatellite instability-high (MSI-H) [3]. The acquisition of these indicators necessitates the collection and analysis of tumor tissue specimens through invasive biopsy procedures, which present challenges in achieving continuous monitoring and longitudinal assessments. Therefore, whether peripheral blood indicators can serve as effective biomarkers has garnered considerable attention from many scientists and clinicians. Selecting peripheral blood as a potential biomarker source has significant scientific value due to its multiple advantages, such as easy collection, minimal risk of complications, reduced impact of tumor heterogeneity, and the ability to reflect the patient’s systemic immune status [4, 5]. These characteristics make peripheral blood an ideal indicator for dynamically monitoring disease progression and drug response kinetics.

Biomarkers associated with tumor burden in peripheral blood, serving as the most direct reflection of neoplastic progression, have historically served as quintessential indicators for evaluating the clinical efficacy of conventional therapeutic regimens in oncology. In 2016, Nicolazzo et al. first demonstrated the connection between PD-L1 positive circulating tumor cells (PD-L1+CTCs) and poorer survival in non-small cell lung cancer (NSCLC) patients treated with nivolumab [6]. Subsequently, in 2017, Cabel et al. were the first to show that quantitative circulating tumor DNA (ctDNA) monitoring could be utilized to evaluate a patient’s response to PD-1 inhibitors [7]. And in 2018, Gandara et al. collected a total of over 1,000 plasma samples from the POPLAR (NCT01903993) and OAK (NCT02008227) clinical trials, and demonstrated that TMB could be accurately and reproducibly measured in plasma and was positively correlated with progression-free survival (PFS) and overall survival (OS) after atezolizumab treatment [8]. In the same year, Kataoka et al. made the report of a serum tumor marker predicting the effectiveness of nivolumab in NSCLC patients, revealing that carcinoembryonic antigen (CEA) levels≥13.8 ng/ml were associated with decreased PFS [9]. The exploration of tumor and tumor-derived biomarkers has continued to deepen.

PD-1/PD-L1 inhibitors can significantly improve the T-cell exhaustion phenotype [10]. Although this immunomodulatory effect is primarily confined within the tumor microenvironment, peripheral blood immune cell subsets also undergo specific dynamic changes due to systemic regulation. These quantifiable features of the peripheral immune system may hold potential as novel biomarkers for predicting treatment response. As early as 2013, Weber et al. performed tetramer staining on leukocytes before and after treatment in 90 advanced melanoma patients receiving nivolumab in a Phase I study, and found that MART-1_26–35_ antigen-specific CD8+ T cells slightly increased at 12 weeks post-treatment in patients with partial response (PR), complete response (CR), and stable disease (SD), but significantly decreased in patients with progressive disease (PD) [11]. Subsequently, lymphocyte count [12] and the derived indicator neutrophil-to-lymphocyte ratio (NLR) [13] have been shown to correlate with better and worse survival after the treatment of PD-1 inhibitors respectively. Technological advancements have enabled researchers to further explore more refined cell subpopulations. From the initial flow cytometry [14], to T cell receptor (TCR)-seq [15], CyTOF detection [16], RNA-seq [17], and scRNA-seq [18], these high-throughput technologies have facilitated the identification of novel peripheral blood biomarkers and demonstrated their significant advantages in this field.

**Fig. 1:**
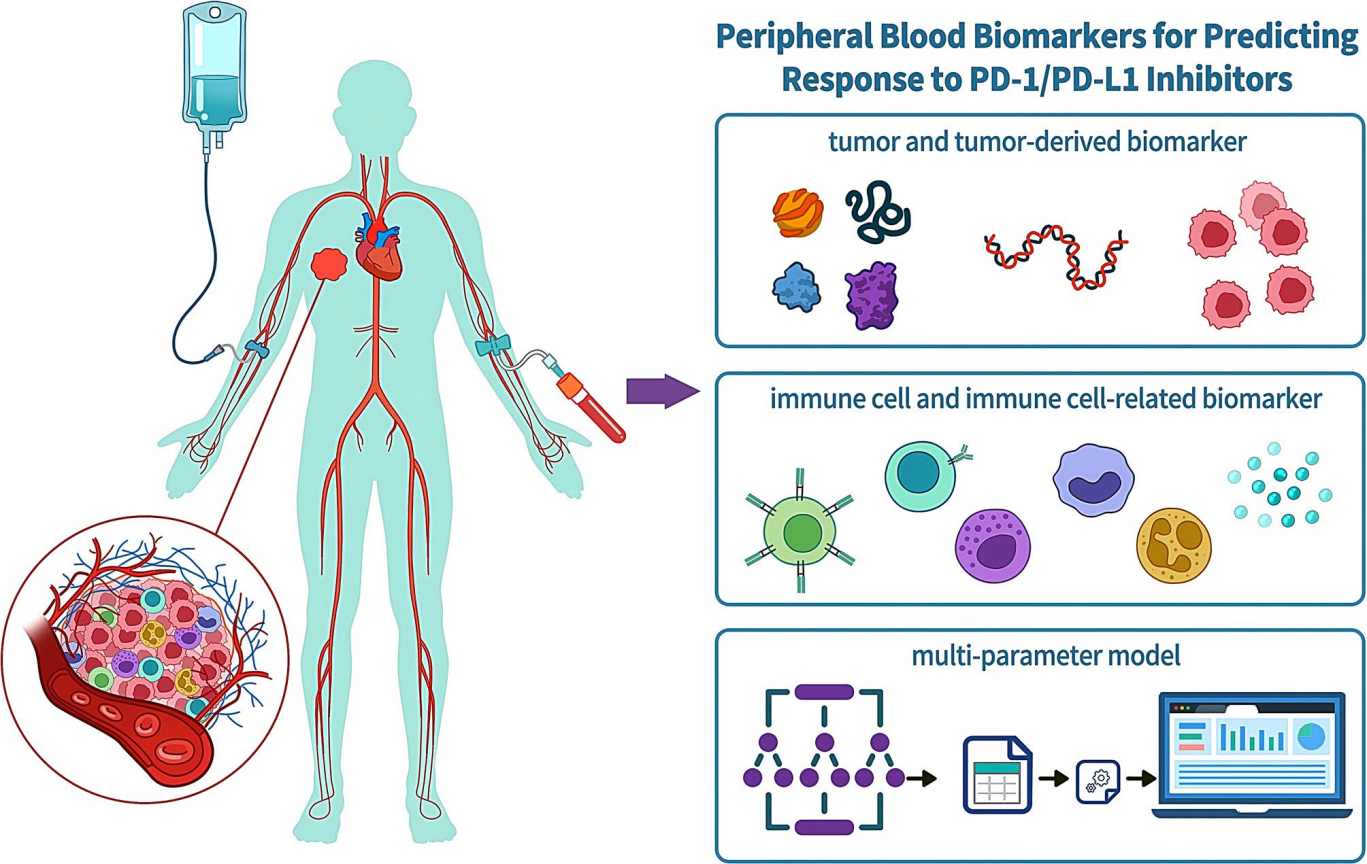
Overview of peripheral blood biomarkers for predicting response to PD-1/PD-L1 inhibitors. Peripheral blood captures both tumor burden and systemic anti-tumor immune activity, supporting its utility as a biomarker source. This review focuses on three major categories: tumor and tumor-derived biomarkers, immune cells and immune cell–related biomarkers, and multi-parameter predictive models, providing a clear framework for the systematic investigation of peripheral blood biomarkers

In view of the current research background and clinical requirements, this review aims to systematically and comprehensively summarize the exploratory findings on the use of peripheral blood samples as biomarkers for predicting the efficacy of anti-PD-1/anti-PD-L1 (aPD-1/aPD-L1) treatment. The summary is organized into three major categories: tumor and tumor-derived biomarkers, immune cell and immune cell-related biomarkers, as well as multi-parameter models (Fig. 1)

## Tumor and tumor-derived biomarkers

Since tumor cells and their derivatives can shed from the primary tumor site and disseminate via the peripheral circulatory system [19], the tumor and tumor-derived biomarkers identified and monitored in peripheral blood tests encompass a variety of types, including tumor-specific markers, circulating tumor cell (CTC), ctDNA, lactate dehydrogenase (LDH), and blood tumor mutation burden (bTMB), etc (Table 1).

**Table 1:** Tumor and tumor-derived markers as predictive biomarkers

Biomarker		Cancer(n)	Treatment		Timepoint	Correlation	Association with clinical outcome		Level*	Ref
			Drug name	ICI type			Response	Survival		
tumor-specific marker	AFP	HCC (*n* = 5322)	none	aPD-1/aPD-L1 mono/combi	baseline	negative	DCR (*p* < 0.001)	PFS (*p* < 0.001) and OS (*p* < 0.001)	3B	[20]
	AFP	HCC(*n* = 77)	(sinti/tisle/camre/pembro/toripa) + Lenvatinib +TACE	aPD-1 + target + chemo	dynamic change	negative		PFS (*p* < 0.001) and OS (*p* = 0.006)	4B	[21]
	CEA	GC(*n* = 105)	(nivo/pembro/sinti/camre/tisle/toripa) mono/+chemo/+chemo +trastuzumab	aPD-1 mono/combi	baseline	negative		PFS (*p* = 0.054) and OS (*p* = 0.038)	3B	[22]
	CEA、CYFRA21-1	NSCLC (*n* = 70)	nivo	aPD-1 mono	dynamic change	negative	DCR(*p* = 0.021，*P* < 0.001)	PFS (*p* = 0.028，*P* < 0.001) and OS (*p* = 0.026，*P* = 0.019)	2A	[23]
	the one with the most prominent change among CEA, CA199, CYFRA21-1 and NSE	NSCLC (*n* = 84)	nivo/pembro/atezo	aPD-1/aPD-L1 mono	dynamic change	negative		PFS (*p* < 0.001) and OS (*p* < 0.001)	4B	[24]
	CEA	NSCLC (*n* = 224)	(tisle/sinti/camre/cadoni/serpluli/nivo/penpuli/tori/zimbere) mono/+target	aPD-1 mono/+target	dynamic change	negative		PFS (*p* = 0.01)	3B	[25]
	CYFRA21-1 < 2.65 ng/ml	NSCLC (*n* = 224)	(tisle/sinti/camre/cadoni/serpluli/nivo/penpuli/tori/zimbere) mono/+target	aPD-1 mono/+target	post-treatment	negative		PFS (*p* = 0.0036)	3B	[25]
ctDNA	tumor-informed maxVAF	UC(*n* = 260)	pembro/gemcitabine with cisplatin or carboplatin	aPD-1 mono	baseline	negative	ORR (pembro arm: *p* = 0.009, chemo arm: *p* > 0.05)	PFS (pembro arm: *p* < 0.001, chemo arm: *p* > 0.05) and OS (pembro arm: *p* < 0.001, chemo arm: *p* > 0.05)	2B	[26]
	5% VAF	GAC(*n* = 43)	nivo+anlotinib hydrochloride	aPD-1+target	baseline	negative		OS (*p* = 0.025)	4A	[27]
	mVAF	RCC(*n* = 12)	nivo mono/+ipi	aPD-1 mono/+aCTLA-4	baseline	negative	DCR (*p* = 0.026)		4A	[28]
	mVAF	STS (*n* = 27)	nivo+ipi	aPD-1+aCTLA-4	baseline	negative	DCR (*p* = 0.02)	PFS (*p* = 0.003) and OS (*p* = 0.004)	4A	[29]
	mVAF	NSCLC (*n* = 262)	(atezo and carboplatin and paclitaxel)/+bevacizumab	aPD-L1+ chemo/aPD-L1+chemo+target	baseline+post-treatment	negative		PFS (*p* < 0.0001)	2B	[30]
	gene mutations	muscle-invasive UC(*n* = 300)	atezo	aPD-L1 mono	baseline	negative		OS (none)	2B	[31]
	gene mutations	muscle-invasive UC(*n* = 99)	atezo	aPD-L1 mono	dynamic change	negative		OS (none)	4A	[31]
	hGE/mL	CHL (*n* = 30)	pembro+AVD chemo	aPD-1+chemo	dynamic change	negative		PFS (*p* = 0.025, *p* = 0.0012)	4A	[32]
	maxVAF	GC(*n* = 30)	(nivo/sinti)+ oxaliplatin/docetaxel/5-fluorouraci/tegafur/capecitabine	aPD-1+chemo	dynamic change	negative	ORR (*p* = 0.0073)	PFS (*p* = 0.003) and OS (*p* = 0.011)	4A	[33]
bTMB	14.5 mut/Mb	NSCLC (*n* = 119)	atezo	aPD-L1 mono	baseline	positive	ORR (*p* < 0.0001)	OS (*p* = 0.032)	2B	[34]
	28mut/Mb^#^	CRC (*n* = 165)	(durva+treme) or BSC	aPD-L1+aCTLA-4	baseline	positive		OS (*p* = 0.02)	2B	[35]
	20mut/Mb^#^	NSCLC (*n* = 809)	(Durva mono/+treme) or platinum-based chemo	aPD-L1 mono/+aCTLA-4	baseline	positive		OS (none)	2B	[36]
	16mut/Mb^#^	HNSCC (*n* = 247)	(Durva mono/+treme) or SoC chemo	aPD-L1 mono/+aCTLA-4	baseline	positive		PFS and OS (none)	2B	[37]
CTC	CTC	NSCLC(*n* = 28)	nivo	aPD-1 mono	baseline	negative		PFS (*p* = 0.02)	4B	[38]
	CTC	NSCLC (*n* = 28)	nivo	aPD-1 mono	post-treatment	negative		PFS (*p* = 0.03)	4B	[38]
	CTC	NSCLC (*n* = 104)	nivo/pembro/atezo	aPD-1/aPD-L1 mono	baseline	negative		PFS (*p* < 0.01) and OS (*p* = 0.05)	2A	[39]
	CTC	NSCLC (*n* = 104)	nivo/pembro/atezo	aPD-1/aPD-L1 mono	post-treatment	negative		PFS (*p* < 0.01) and OS (*p* < 0.01)	2A	[39]
	CTC	NSCLC (*n* = 83)	pembro/atezo	aPD-1/aPD-L1 mono	dynamic change	negative		PFS (*p* = 0.078) and OS (*p* = 0.021)	2A	[40]
	CTC	HER2+EGA (*n* = 63)	(nivo and trastuzumab) +ipi/FOLFOX	(aPD-1+target)+chemo/aCTLA-4	post-treatment	negative		OS (ipi arm: *p* = 0.0041, FOLFOX:*p* = 0.52)	4A	[41]
	CTC	G/GEJ adenocarcinoma (*n* = 38)	neo tisle+SOX chemo	aPD-1+chemo	post-treatment	positive		PFS (*p* = 0.0035)	4A	[42]
	PD-L1+ CTC	NSCLC (*n* = 96)	nivo	aPD-1 mono	baseline	negative		PFS (*p* = 0.04)	2A	[43]
	PD-L1+ CTC	Melanoma (*n* = 25)	pembro	aPD-1 mono	baseline	positive		PFS (*p* = 0.018)	4A	[44]
	PD-L1+ CTC	NSCLC (*n* = 30)	sinti+ docetaxel	aPD-1+chemo	baseline	positive	ORR (*p* = 0.021)	PFS (*p* = 0.011) and OS (*p* = 0.038)	4A	[45]
LDH	ULN	Melanoma (*n* = 935)	nivo mono/+ipi	aPD-1 mono/+aCTLA-4	baseline	negative	ORR (*p* = 0.023，*P* = 0.0002)		3B	[46]
	ULN and 2ULN	Melanoma (*n* = 207)	nivo/pembro	aPD-1 mono	baseline	negative		OS (*p* < 0.0001)	3B	[47]
	265 U/L	PAAD (*n* = 67)	(toripa/sinti/pembro)+chemo/radio/target	aPD-1 combi	baseline	negative		PFS (*p* < 0.001) and OS (*p* < 0.001)	4B	[48]
	220 U/L	NSCLC (*n* = 224)	(tisle/sinti/camre/cadoni/serpluli/nivo/penpuli/tori/zimbere) mono/+target	aPD-1 mono/+target	baseline	negative		PFS (*p* = 0.0008)	3B	[25]

Biomarker: *AFP* Alpha-fetoProtein, *CEA* carcinoembryonic antigen, *CYFRA21-1* cytokeratin 19 fragment antigen21 -1, *CA199* carbohydrate antigen 19-9, *NSE* neuron-specific enolase, *ctDNA* circulating tumor DNA, *mVAF* median variant allelic frequency, *hGE* haploid genome equivalent, *bTMB* blood tumor mutation burden, *CTC* circulating tumor cell, *LDH* lactate dehydrogenase, *ULN* upper limit of normal

Cancer: *HCC* hepatocellular carcinoma, *GC* gastric cancer, *NSCLC* non-small-cell lung cancer, *UC* urothelial carcinoma, *GAC* gastric adenocarcinoma, *RCC* renal cell carcinoma, *STS* soft tissue sarcomas, *CHL* classic Hodgkin lymphoma, *CRC* colorectal cancer, *HNSCC* head and neck squamous cell carcinoma, *HER2+ EGA* HER2-positive esophagogastric adenocarcinoma, *G/GEJ* adenocarcinoma gastric and gastroesophageal junction adenocarcinoma, *PAAD* pancreatic adenocarcinoma

Treatment: *TACE* transarterial chemoembolization, *AVD* doxorubicin, vinblastine and dacarbazine, *BSC* best supportive care, *SoC* chemo cetuximab, a taxane, methotrexate, or a fluoropyrimidine, *FOLFOX* folinic acid, fluorouracil and oxaliplatin, *SOX* S-1 and oxaliplatin

Clinical outcome: *DCR* disease control rate, *PFS* progression-free survival, *OS* overall survival, *ORR* objective response rate

# indicates the comparison of benefits from different treatment methods

* Evidence levels: Level 1 Evidence based on prospective randomized controlled trials (RCTs): Pre-specified analyses from large prospective RCTs demonstrating that biomarker-guided treatment strategies significantly improve clinical outcomes (1A) or meta-analyses of multiple high-quality RCTs with pre-specified biomarker analyses (1B). Level 2 Prospective analyses: Prospective cohort studies for biomarker validation (2A) or exploratory subgroup analyses within prospective single-arm trials or prospective cohorts (sample size ≥ 100) (2B). Level 3 Large retrospective biomarker analyses (sample size ≥ 100) with (3A) or without (3B) validation cohorts. Level 4: Small exploratory biomarker analyses (sample size < 100), either prospective (4A) or retrospective (4B). Level 5 Hypothesis-generating studies, including case reports, case series, or preclinical research (cell-based or animal studies)

### Commonly used tumor-specific markers in clinical practice

Serum tumor-specific markers, which indicate the existence and proliferation of neoplasms, have been demonstrated to correlate with adverse therapeutic outcomes in studies involving conventional chemotherapy [49–51], radiotherapy [51–53] and targeted therapy [54, 55], et al. Furthermore, the predictive value of these markers remains evident in studies utilizing aPD-1/aPD-L1-based treatments (Table 1). A retrospective analysis involving 44 articles and 5,322 hepatocellular carcinoma (HCC) patients treated with aPD-1/aPD-L1 monotherapy or combination therapies showed that patients with lower baseline Alpha-fetoProtein (AFP) levels exhibited higher disease control rate (DCR), as well as longer PFS and OS [20]. In the study by Luo et al. (*n* = 77), HCC patients who experienced a reduction in AFP levels after treatment also exhibited longer PFS and OS [21]. Additionally, a retrospective study including 105 patients with metastatic gastric cancer (GC) indicated that higher baseline CEA levels were associated with worse PFS and OS in patients receiving aPD-1 monotherapy or combination therapy [22]. Moreover, multiple studies involving NSCLC patients treated with various aPD-1/aPD-L1-based therapies also observed a negative correlation between post-treatment CEA and cytokeratin 19 fragment antigen21-1(CYFRA21-1) levels and PFS or OS [23–25].

Current research focuses on cancers with specific markers like NSCLC, GC, and HCC. According to evidence grading, only the study by Dal Bello et al. which prospectively verified the correlations between CEA, CYFRA21-1 and the efficacy of nivolumab, demonstrates Level 2A evidence [23], all others are Level 3B or 4B, confining the field to an exploratory phase reliant on retrospective cohorts and highlighting the need for prospective validation (See Table 1 for evidence-level grading of the included studies).

### ctDNA

The acquisition of ctDNA derived from tumors is more feasible compared to CTCs, thus making ctDNA the most widely used tumor-derived genomic biomarker in research. Moreover, ctDNA not only serves as a surrogate marker of tumor burden but also facilitates the detection of immune-related genetic alterations [56, 57], thereby offering insights into mechanisms of resistance to immune checkpoint inhibitors (ICIs). These attributes underscore the considerable potential of ctDNA as a predictive biomarker for aPD-1/aPD-L1 therapeutic efficacy. In March 2024, the RECIST Working Group proposed a standardized plan for the liquid biopsy response evaluation criteria in solid tumors (LB-RECIST), aiming to facilitate the clinical application of assessing treatment response through dynamic changes in ctDNA [58].

Baseline ctDNA levels have demonstrated a significant negative correlation with treatment response and survival outcomes in patients with various cancers receiving aPD-1/aPD-L1 monotherapy or combination therapies [26–29, 31]. Ding et al. conducted ctDNA testing on the peripheral blood of 262 NSCLC patients receiving atezolizumab combined with chemotherapy with or without bevacizumab at baseline and during the subsequent four treatment cycles [30]. They found that using the median variant allelic frequency (mVAF) of ctDNA across all samples as the cutoff value, patients with the lowest ctDNA VAF below the cutoff during the entire treatment course exhibited longer PFS [30]. Some studies have focused more on the clearance efficiency of ctDNA. For example, Powles et al. reported the updated survival data based on ctDNA status in the Phase III IMvigor010 trial [31]. They found that a greater reduction in ctDNA levels after two cycles of atezolizumab treatment was associated with longer OS (100% clearance, 60.0 months; 50–99% reduction, 34.3 months; < 50% reduction, 19.9 months) [31]. A positive correlation between ctDNA clearance and PFS was also observed in patients with classical Hodgkin lymphoma (*n* = 30) [32] and GC (*n* = 30) [33] treated with aPD-1 combined with chemotherapy (See Table 1 for details).

The evidence grading of ctDNA-related studies indicates that three prospective phase III RCTs have conducted exploratory analyses involving ctDNA [26, 30, 31], providing Level 2B evidence. Although the other six studies are classified as small-scale exploratory analyses, they are still conducted within prospectively designed cohorts (Level 4A). The consistent, strong negative association between ctDNA and immunotherapy efficacy, as demonstrated across current studies, supports its predictive utility. The next essential step is its validation in prospective RCTs or large-scale cohorts to attain the Level 1-2A evidence necessary for clinical application.

### bTMB

Previous retrospective and prospective research has identified tumor-based TMB as a predictive biomarker of response to ICIs [59], and based on the findings of the KEYNOTE-158 clinical trial, the FDA approved TMB as a companion diagnostic biomarker for pembrolizumab for the first time in 2020 [4]. Due to the positive correlation between bTMB and tumor-based TMB [60], researchers are also actively exploring the use of bTMB as a substitute for TMB.

As expected, high bTMB has been confirmed to be associated with prolonged survival in multiple clinical trials (Table 1). B-F1RST trial (Phase II) evaluated whether bTMB could serve as a predictive biomarker for first-line atezolizumab monotherapy in 119 patients with locally advanced or metastatic NSCLC, and found that patients with bTMB ≥ 14.5 mut/Mb had higher objective response rate (ORR) and OS [34]. In addition, CO.26 trial (Phase II) demonstrated that colorectal cancer patients treated with durvalumab plus tremelimumab (D+T) (*n* = 118) exhibited longer OS compared to those receiving best supportive care (BSC) (*n* = 50) when bTMB ≥ 28 mut/Mb [35]. In the MYSTIC study (Phase III, *n* = 809), it was determined that patients with bTMB ≥ 20 mut/Mb showed improved OS for durvalumab plus tremelimumab vs chemotherapy (21.9 months vs 10.0 months) [36]. Similarly, in the EAGLE study (Phase III, *n* = 247), a significant OS benefit versus chemotherapy was observed with durvalumab and durvalumab plus tremelimumab at bTMB≥16 mut/Mb respectively in recurrent or metastatic head and neck squamous cell carcinoma (HNSCC) patients [37].

Across multiple studies, bTMB is supported by consistent Level 2B evidence, underscoring its strong potential for clinical translation as a biomarker of tumor immunogenicity and a promising complement to PD-L1. Despite this promise, substantial variability in applied thresholds poses a key challenge. Moving forward, it is imperative to harmonize testing methodologies and establish standardized cutoffs to ensure reliable comparison across studies and facilitate its integration into routine clinical practice.

### CTC

In principle, CTC can serve as a marker for tumor burden across all cancer types, as they are released into the bloodstream for detection [56, 61]. Several studies have also demonstrated a significant negative correlation between baseline or post-treatment CTC levels and PFS or OS in NSCLC patients receiving aPD-1/aPD-L1 monotherapy [38–40]. Similarly, in the INTEGA clinical trial (*n* = 63), HER2-positive esophagogastric adenocarcinoma patients who had no detectable CTCs after one cycle of nivolumab combined with trastuzumab and ipilimumab treatment demonstrated significantly longer OS [41].

However, Sun et al. reported that in 38 patients with locally advanced gastric and gastroesophageal junction adenocarcinoma treated with neoadjuvant tislelizumab combined with chemotherapy, patients with preoperative CTC counts ≥3 had significantly longer PFS compared to those with CTC < 3, suggesting a favorable impact of CTCs, which contrasts with the findings of previous studies [42]. This heterogeneity in outcomes may be linked to the heterogeneity of CTCs, particularly regarding the tumor cell PD-L1 expression, which can predict response to aPD-1/aPD-L1 therapy. Guibert et al. found that NSCLC patients (*n* = 96) with PFS < 6 months had a higher baseline PD-L1+ CTC value (≥1%) during nivolumab treatment [43], whereas PD-L1+ CTCs showed a positive correlation with PFS in melanoma patients treated with pembrolizumab (*n* = 25) [44] and NSCLC patients treated with sintilimab combined with docetaxel (*n* = 30) [45].

Higher-level evidence (including Level 2A) consistently supports the negative correlation between CTCs (and PD-L1+CTCs) and treatment efficacy, in contrast to the limited, lower-level (Level 4A) evidence for positive correlations (Table 1). This preliminarily validates the predictive value of CTCs, though resolving heterogeneity related to PD-L1+CTCs remains a key challenge for future studies.

### LDH

Serum LDH is considered to originate from tumor necrosis and lysis as well as normal cell damage caused by tumors, which can serve as a potential indicator of tumor burden [62, 63]. Moreover, it was reported that LDH-derived lactate can upregulate the expression of PD-L1 on tumor cells [64], and blockade of LDH improves the efficacy of aPD-1 therapy in melanoma [65]. Consequently, LDH represents a highly competitive candidate as a predictive biomarker for treatment response to ICIs.

Overall, in studies evaluating both monotherapy and combination therapy, elevated levels of LDH have been associated with poor efficacy and shortened survival time. A retrospective analysis integrating three studies—CheckMate 069 (nivo+ipi = 95), CheckMate 066 (nivo+ipi = 314, nivo = 316), and CheckMate 067 (nivo = 210), with a total of 935 patients with advanced melanoma showed that baseline LDH levels above the upper limit of normal (ULN) were associated with poorer ORR in both the nivolumab plus ipilimumab group (*n* = 409) and the nivolumab monotherapy group (*n* = 526) [46]. In another study involving melanoma patients (*n* = 207) treated with nivolumab/pembrolizumab, the 24-month OS rate was highest for patients with normal LDH levels at 39%, compared to 19% for those with LDH > ULN and only 8% for those with LDH > 2ULN [47]. Furthermore, in pancreatic cancer patients (*n* = 67) [48]and NSCLC patients (*n* = 224) [25] receiving various treatments based on aPD-1 therapy, researchers identified thresholds of 265 U/L and 220 U/L respectively, with patients below the threshold showing significantly longer PFS (See Table 1 for details).

Thus, the current body of evidence, while consistently highlighting LDH's predictive value, is defined by its retrospective nature (Level 3B/4B) and unresolved methodological heterogeneity. Future efforts must therefore focus on threshold standardization and prospective validation to assess its true clinical potential.

Across biomarker dimensions—from molecular (tumor-specific markers, ctDNA, bTMB) and phenotypic (CTCs) to systemic (LDH)—predictive potential is consistently constrained by standardization issues in thresholds and phenotyping. Future translation hinges on their unified validation within prospective, large-scale cohorts to consolidate clinical evidence.

Classical tumor-derived biomarkers are increasingly viewed not merely as passive indicators but as dynamic elements that appear to shape the tumor-immune dialogue and ICI efficacy. CEA levels have been shown to negatively correlate with tumor-infiltrating T-cell density in colorectal cancer [66]. As molecular features of the tumor, both ctDNA and bTMB provide insights that extend beyond burden and immunogenicity to encompass immune interaction. The synchronized kinetics between ctDNA decline and T-cell expansion in responders [67], while the neoantigen burden reflected by bTMB must be coupled with competent major histocompatibility complex (MHC)-mediated presentation and a diverse T TCR repertoire for effective immune targeting [68]. The role of CTCs extends beyond representing tumor burden and aggressiveness; they may directly inhibit T and natural killer (NK) cell activation via surface expression of immune checkpoints (PD-L1, CTLA-4, CD47) and indirectly suppress immunity by secreting factors that recruit immunosuppressive myeloid-derived suppressor cells (MDSCs) and regulatory T cells (Tregs) [69]. Beyond its established role as a systemic biomarker of tumor burden, LDH also functions as a potent immunosuppressive agent. It acts by suppressing key cytotoxic T-cell mediators (IFN-γ, perforin, granzyme), impeding dendritic cell maturation, weakening NK cell cytotoxicity, and inducing Treg differentiation, thereby establishing a broadly immunosuppressive phenotype [62, 70]. These factors are intrinsically linked to antitumor immunity, with their synergy being central to its core mechanisms, underscoring the need to integrate immune parameters to refine therapeutic strategies against high tumor burden.

## Immune cells and immune cell-related biomarkers

Early exploratory clinical trials have shown that tumor-specific T cells in the peripheral blood of patients who respond to treatment were effectively activated after ICI therapy [11]. However, due to the limitations in detection methods, its widespread application has been restricted, making it urgent to explore other related biomarkers.

### Lymphocyte count and NLR

Lymphocyte count and its derivative index NLR can be obtained through a simple blood routine test, which show strong application potential and represent some of the earliest applied markers [71] (Table 2). In a prospective study involving 134 NSCLC patients treated with nivolumab, patients with baseline absolute lymphocyte count (ALC) ≥ 1000/μL exhibited significantly better PFS and OS [72]. Subsequently, Lee et al. also confirmed in NSCLC patients (*n* = 231) receiving various aPD-1/aPD-L1 monotherapies that high ALC levels before and after treatment were associated with better ORR, PFS, and OS [73]. Research on NLR has been more extensive, showing a consistent negative correlation with efficacy across different treatment modalities. In NSCLC (*n* = 157) [74], melanoma (*n* = 97) [75], and esophageal squamous cell carcinoma(ESCC) (*n* = 81) [76] patients treated with aPD-1 monotherapy, those with lower baseline NLR exhibited better OS, but the cutoff values of NLR derived from these studies varied, which were 5.9, 5, and 3, respectively. In NSCLC patients (*n* = 116) receiving neoadjuvant aPD-1 combined with chemotherapy, both baseline NLR < 3.05 and preoperative NLR < 2.47 were associated with longer disease-free survival (DFS) [77]. Moreover, cutoff values of NLR < 2.4 and NLR < 5 were respectively identified in HCC patients receiving sintilimab combined with tyrosine kinase inhibitors (*n* = 49) [78] and atezolizumab combined with bevacizumab (*n* = 120) [79]. Meanwhile, Qiu et al. also found that among advanced pancreatic cancer patients (*n* = 67) receiving various aPD-1-based treatments, those with baseline NLR≤2 achieved higher ORR and DCR [48].

**Table 2:** Lymphocyte count and NLR as predictive biomarkers

Biomarker	Cancer(n)	Treatment		Timepoint	Correlation	Association with clinical outcome		Level*		Ref
		Drug name	ICI type			Response	Survival			
ALC≥1000/μL	NSCLC(*n* = 134)	nivo	aPD-1 mono	baseline	positive		PFS (*p* < 0.04) and OS (*p* < 0.03)	3B	[72]	
ALC	NSCLC(*n* = 231)	nivo/pembro/atezo	aPD-1/aPD-L1 mono	baseline	positive	ORR (*p* = 0.02)	PFS (*p* = 0.006) and OS (*p* = 0.035)	3B	[73]	
ALC	NSCLC(*n* = 231)	nivo/pembro/atezo	aPD-1/aPD-L1 mono	post-treatment	positive	ORR (*p* = 0.02)	PFS (*p* = 0.001) and OS (*p* = 0.005)	3B	[73]	
NLR < 5.9	NSCLC(*n* = 157)	nivo/pembro	aPD-1 mono	baseline	negative		OS (*p* = 0.004)	3B	[74]	
NLR < 5	Melanoma(*n* = 97)	nivo	aPD-1 mono	baseline	negative		PFS (*p* < 0.0001) and OS (*p* < 0.0001)	4B	[75]	
NLR < 3	ESCC (*n* = 81)	sinti	aPD-1 mono	baseline	negative		PFS (*p* = 0.002) and OS (*p* = 0.019)	4A	[76]	
NLR < 3	ESCC (*n* = 65)	sinti	aPD-1 mono	post-treatment	negative		PFS (*p* = 0.006) and OS (*p* < 0.001)	4A	[76]	
NLR < 3.05	NSCLC(*n* = 116)	neo (pem/nivo/camre/tori/tisle/sinti)+cisplatin/carboplatin+pemetrexed/paclitaxel	aPD-1+chemo	baseline	negative		DFS (*p* = 0.021)	3B	[77]	
NLR < 2.47	NSCLC(*n* = 116)	neo (pem/nivo/camre/tori/tisle/sinti)+cisplatin/carboplatin+pemetrexed/paclitaxel	aPD-1+chemo	post-treatment	negative		DFS (*p* = 0.004)	3B	[77]	
NLR < 2.4	HCC(*n* = 49)	sinti+TKI(lenvatinib/sorafenib)	aPD-1+target	baseline	negative		OS (*p* = 0.01)	4B	[78]	
NLR < 5	HCC(*n* = 120)	atezo+bevacizumab	aPD-L1+target	baseline	negative		PFS (*p* = 0.023) and OS (*p* < 0.001)	3B	[79]	
NLR≤2	PAAD (*n* = 67)	(toripa/sinti/pembro)+chemo/radio/target	aPD-1 combi	baseline	negative	ORR (*p* = 0 0.037) and DCR (*p* = 0.032)		4B	[48]	

Biomarker: *ALC* absolute lymphocyte count, *NLR* neutrophil-to-lymphocyte ratio

Cancer: *NSCLC* non-small-cell lung cancer, *ESCC* esophageal squamous cell carcinoma, *HCC* hepatocellular carcinoma, *PAAD* pancreatic adenocarcinoma

Treatment: *neo* neoadjuvant, *TKI* tyrosine kinase inhibitor

Clinical outcome: *PFS* progression-free survival, *OS* overall survival, *ORR* objective response rate, *DFS* disease-free survival, *DCR* disease control rate

* Evidence levels: defined as in 1

Except for the finding of NLR < 3 in ESCC [76], which is derived from a prospective study and classified as Level 4A evidence, all other studies investigating ALC and NLR are based on retrospective cohorts, corresponding to Level 3B or 4B evidence. These results indicate that this category of biomarkers remains largely at the stage of retrospective exploration. The significant variation in the reported optimal cutoff values across studies may be due to the large variation in the NLR value itself in tumor patients under basic conditions [80, 81]. This heterogeneity may hinder the establishment of a universal cutoff for NLR, highlighting the need to explore its optimal application conditions in specific clinical contexts or to enhance its predictive accuracy through combination with other biomarkers.

### T cells

#### CD8+T

CD8+T cells, as the primary cytotoxic cells in anti-tumor cellular immunity [82], are considered one of the most promising biomarkers. With the advancement of high-throughput technologies, researchers have supplemented markers for naïve, cytotoxic, and memory states of T cells and further identified markers for senescence, exhaustion/dysfunction, and other functional phenotypes [83]. However, due to the cost of technologies and time constraints, flow cytometry remains the primary method for studying T cell phenotypes in peripheral blood [71], and might also become the mainstream technology for future clinical applications. At present, numerous studies have reported the correlation between peripheral blood CD8+ T cells and the response to aPD-1/aPD-L1 therapy (Table 3).

**Table 3:** CD8+T cells as predictive biomarkers

Biomarker	Cancer(n)	Treatment		Timepoint	Correlation	Association with clinical outcome		Level*	Ref
		Drug name	ICI type			Response	Survival		
CD8+T	MPLC (*n* = 36)	sinti	aPD-1 mono	baseline	positive	ORR (*p* < 0.001)		4A	[84]
PD-1+CD8+T	GC (*n* = 19)	pembro	aPD-1 mono	baseline	positive		PFS (*p* = 0.0435)	4A	[85]
CD103+PD-1+CD8+T	GC (*n* = 29)	nivo	aPD-1 mono	post-treatment	positive		PFS (*p* = 0.032)	4A	[86]
CD103+CD39+ CD8+ T	NSCLC (*n* = 21)	nivo/pembro/atezo/durva	aPD-1/aPD-L1 mono	dynamic change	positive		PFS (*p* ≤ 0.01)	4A	[87]
PD-1+TIGIT+CD8+T	Melanoma (*n* = 26)	nivo/pembro	aPD-1 mono	post-treatment	positive	ORR (*p* < 0.05)	OS (*p* = 0.0019)	4A	[88]
CD73+PD-1+CD8+T	Melanoma (*n* = 100)	nivo	aPD-1 mono	baseline	negative	CBR (6 months) (*p* = 0.02)	OS (*p* = 0.001)	2A	[89]
PD-1+Eomes+CD8+T	NSCLC (*n* = 54)	nivo	aPD-1 mono	baseline	negative	DCR (*p* = 0.046)		4A	[90]
CD28–CD57+KLRG1+CD8+T	NSCLC (*n* = 83)	none	aPD-1/aPD-L1 mono	baseline	negative	ORR (*p* = 0.002)	PFS (*p* < 0.0001) and OS (*p* < 0.007)	4A	[91]
CCR7+GZMK+PD-1-TIGIT-CD8+T	pleural mesothelioma (*n* = 35)	durva+ pemetrexed +cisplatin	aPD-L1+chemo	baseline	positive		PFS (*p* = 0.0076) and OS (*p* = 0.021)	4A	[92]
CX3CR1+CD8+ T	NSCLC (*n* = 29)	pembro/atezo+carboplatin+pemetrexed/paclitaxel	aPD-1/aPD-L1+chemo	dynamic change	positive	ORR (*p* < 0.0001)	PFS (*p* = 0.0051) and OS (*p* = 0.0138)	4A	[93]
LAG3+PD-1+CD8+T	CRC(*n* = 30)	pembro+FOLFOX	aPD-1+chemo	baseline	negative	ORR (*p* = 0.0379)	PFS (*p* = 0.0262)	4A	[94]
CD62L+ CD45RA-CD3+ CD8+T	SCLC (*n* = 32)	atezo+ carboplatin and etoposide	aPD-L1+chemo	dynamic change	negative		OS (*p* = 0.044)	4A	[95]
CD8+T	ACC (*n* = 21)	camre + apatinib	aPD-1+target	baseline	positive	ORR (*p* = 0.002)		4A	[96]
Ki-67+PD-1+CD8+T	HCC(*n* = 29)	atezo+bevacizumab	aPD-L1+target	baseline	positive		OS(*p* = 0.011)	4A	[97]
PD-L1+ CD8+T	HCC(*n* = 37)	atezo+bevacizumab	aPD-L1+target	baseline	negative	DCR (*p* = 0.01)		4A	[98]
CD8+T	Melanoma (*n* = 28)	atezo and cobimetinib/+vemurafenib	aPD-L1+target	baseline	positive	pCR and near pCR(*p* = 0.02)		4A	[99]
CD8+T	Melanoma (*n* = 28)	atezo and cobimetinib/+vemurafenib	aPD-L1+target	post-treatment	positive	pCR and near pCR (one cycle, *p* = 0.03; four cycles, *p* = 0.04)		4A	[99]
Ki-67+CD8+ T and Ki-67+PD-1+CD8+ T	HCC(*n* = 42)	nivo+regorafenib	aPD-1+target	dynamic change	positive	CR/PR > 10 months (*p* = 0.313, *p* = 0.313)		4A	[100]
GZMB+perforin+ CD8+T and GZMB+perforin+PD-1+ CD8+T	HCC(*n* = 42)	nivo+regorafenib	aPD-1+target	dynamic change	positive	CR/PR > 10 months (*p* = 0.469, *p* = 0.313)		4A	[100]
GZMB+CD8+T	NSCLC (*n* = 38)	nivo mono/+relatlimab	aPD-1 mono/+ aLAG-3	dynamic change	positive	MPR (*p* = 0.0002)		4A	[101]
CD45RA+CCR7+CD8+T	pleural mesothelioma (*n* = 62)	nivo mono/+ipi	aPD-1 mono/+aCTLA-4	baseline	positive		PFS (*p* = 0.045) and OS(*p* = 0.086)	4A	[102]
CD8+CD28+T	pan-cancer (*n* = 87)	(pem/nivo/camre/tori/tisle/sinti/durva/atezo/penpulimab)+chemo/target/ipi	aPD-1/aPD-L1 combi	baseline	positive	ORR (*p* < 0.001)	PFS (*p* < 0.001) and OS (*p* = 0.004)	4B	[103]
HLA-DR+CD38+CD8+T	RCC (*n* = 36)	nivo、nivo+ipi 、nivo+bempegaldesleukin(IL-12)	aPD-1 combi	dynamic change	positive	the reduced volume of the tumor (*p* = 0.021)	PFS (*p* = 0.006)	4A	[104]
HVEM+CD8+T and CD69+CD8+T	Melanoma (*n* = 29)	nivo+BRAFi ± MEKi/nivo++ipi	aPD-1 combi	post-treatment	negative		PFS (*p* < 0.0001，*P* < 0.05)	4A	[105]

Cancer: *MPLC* multiple primary lung cancer, *GC* gastric cancer, *NSCLC* non-small-cell lung cancer, *CRC* colorectal cancer, *SCLC* small cell lung cancer, *ACC* adrenal cortical carcinoma, *HCC* hepatocellular carcinoma, *RCC* renal cell carcinoma

Treatment: FOLFOX folinic acid, fluorouracil and oxaliplatin

Clinical outcome: *ORR* objective response rate, *PFS* progression-free survival, *OS* overall survival, *CBR* clinical benefit rate, *PR* partial response, *CR* complete response, *DCR* disease control rate, *pCR* pathological complete response, *MPR* major pathological response

* Evidence levels: defined as in Table 1

##### APD-1/aPD-L1 monotherapy

In studies where aPD-1/aPD-L1 monotherapy was used as the treatment approach, a Phase II clinical trial including 36 multiple primary lung cancer patients treated with sintilimab showed that responders (CR/PR patients) exhibited a higher baseline proportion of CD8+T (CD45+CD3+CD8+T) cells, and maintained higher CD8+T levels than non-responders over three treatment cycles [84]. Many studies have further explored CD8+ T cells with specific markers. Previous studies have shown that baseline PD-1+CD8+ T cells [85] and post-treatment CD103+PD-1+CD8+ T cells [86] are positively correlated with longer PFS in GC patients (*n* = 19 and *n* = 29) receiving aPD-1 monotherapy. Co-expression of CD103 and CD39 has been demonstrated to identify tumor-reactive CD8+ T cells within the tumor microenvironment [106]. Thus, in NSCLC patients (*n* = 21) treated with various aPD-1/aPD-L1 monotherapies, those with a greater increase in peripheral blood CD103+CD39+CD8+ T cell levels post-treatment also exhibited longer PFS [87]. Additionally, CD8+ T cells co-expressing PD-1 and TIGIT after treatment were found to be associated with better response and OS in melanoma patients (*n* = 26) treated with nivolumab/pembrolizumab [88]. However, Capone et al. evaluated the correlation between CD73+PD-1+CD8+ T cells (adenosine derived from CD73 can inhibit anti-tumor immunity) and nivolumab efficacy in 100 melanoma patients [89]. They found that patients with durable clinical benefit (CR/PR/SD > 6 months) exhibited lower baseline levels of CD73+PD-1+CD8+ T cells, and patients with this cell population < 2.3% had longer OS [89]. In NSCLC patients, exhausted phenotype PD-1+Eomes+CD8+ T cells [90] and senescent phenotype CD28–CD57+KLRG1+CD8+ T cells [91] also showed a negative correlation with responses to aPD-1/aPD-L1 monotherapy.

##### The combination of aPD-1/aPD-L1 and chemotherapy

In studies employing the combination of aPD-1/aPD-L1 and chemotherapy as the treatment approach, Chin et al. identified a population of stem cell-like T cells (CCR7+GZMK+PD-1-TIGIT-CD8+T) in the peripheral blood of pleural mesothelioma patients (*n* = 35) treated with durvalumab in combination with pemetrexed and cisplatin [92]. Patients with higher enrichment of these cells exhibited significantly longer PFS and OS [92]. Additionally, Abdelfatah et al. found that NSCLC patients (*n* = 29) treated with pembrolizumab/atezolizumab combined with chemotherapy, who showed a ≥ 10% increase in CX3CR1+CD8+T cells post-treatment, had better ORR, PFS, and OS [93]. Similarly, some CD8+T cells demonstrated a negative correlation with efficacy. In colorectal cancer (CRC) patients (*n* = 30) receiving pembrolizumab combined with FOLFOX6, CR/PR patients had lower baseline levels of LAG3+PD-1+CD8+T cells, and patients below the median level of LAG3+PD-1+CD8+T cells exhibited better PFS [94]. Meanwhile, Herting et al. found that in small cell lung cancer (SCLC) patients (*n* = 32) treated with atezolizumab combined with carboplatin and etoposide, patients who experienced a reduction in central memory CD8+T cell (CD62L+ CD45RA-CD3+ CD8+T) after two treatment cycles had longer cumulative survival [95].

##### The combination of aPD-1/aPD-L1 and other targeted therapies

In addition to chemotherapy, combining ICIs with targeted therapies is also an important treatment approach for certain cancer types, including combinations with angiogenesis inhibitors, tumor small-molecule targeted therapies, and multi-target tyrosine kinase inhibitors [107]. In studies exploring the combination of aPD-1/aPD-L1 and angiogenesis inhibitors, Zhu et al. found a positive correlation between baseline CD8+ T cells and response to treatment (CR/PR) in adrenal cortical carcinoma patients treated with camrelizumab and apatinib (*n* = 21) [96]. In HCC patients treated with atezolizumab plus bevacizumab, Ki-67+PD-1+CD8+ T cells showed a positive correlation with OS in Lee et al.‘s study (*n* = 29) [97], whereas PD-L1+CD8+ T cells were significantly negatively correlated with treatment response in Gramantieri et al.‘s study (*n* = 37) [98]. In research on tumor small-molecule targeted therapy combinations, the Phase II NeoACTIVATE clinical trial (30 melanoma patients receiving atezolizumab plus cobimetinib with or without vemurafenib, among which 28 patients have blood samples) demonstrated that patients achieving pathological complete response (pCR) or near-pCR (tumor cell proportion ≤10%) had significantly higher levels of CD8+ T cells before treatment, after one cycle of treatment, and after four cycles of treatment compared to other patients [99]. In the same year, the Phase II RENOBATE trial (42 HCC patients received nivolumab combined with regorafenib) observed an increase in the proportion of Ki-67+ cells and GZMB+ perforin+ cells in CD8+ T cells and PD-1+CD8+ T cells only in patients with CR/PR > 10 months after treatment [100].

##### The combination of aPD-1/aPD-L1 and other ICIs

When PD-1/PD-L1 inhibitors are combined with other ICIs, certain cell populations exhibit similar effects in both monotherapy and combination therapy. For instance, Schuler et al. reported in a study of 38 NSCLC patients treated with nivolumab alone or in combination with relatlimab that a significant increase in the proportion of GZMB+CD8+ T cells was observed only in major pathological response (MPR) patients (tumor cells ≤ 50%) after four weeks of treatment (*p* = 0.0002), and the effect was comparable in both monotherapy (*p* = 0.04) and combination therapy (*p* = 0.068) [101]. However, Mankor et al. found that in malignant pleural mesothelioma patients treated with nivolumab alone or in combination with ipilimumab, high baseline levels of CD45RA+CCR7+CD8+ T cells were significantly associated with longer PFS only in the combination therapy group (*n* = 32) (*p* = 0.045), with a similar trend for OS but no statistical difference, while this phenomenon was absent in the monotherapy group (*n* = 23) [102]. These results suggest that aPD-1/aPD-L1 monotherapy and its combination with other ICIs may have distinct predictive biomarkers of efficacy due to differences in their mechanisms of action, which requires further exploration.

##### Various aPD-1/aPD-L1-based treatments

Some studies have simultaneously included patients receiving various treatment modalities based on aPD-1/aPD-L1, aiming to identify potentially universal biomarkers. For example, Geng et al. retrospectively analyzed 87 patients with various cancers treated with aPD-1/aPD-L1 in combination with chemotherapy, targeted therapy, or other ICI treatments, and classified them into three groups according to baseline CD8+CD28+ T cell levels (excessive: ≥ 309/μL; high: 309–190/μL; low: < 190/μL). The results showed that patients in the excessive or high groups had significantly higher ORR, PFS, and OS [103]. Additionally, among renal cell carcinoma (RCC) patients (*n* = 36) receiving multiple aPD-1 monotherapy or combination treatments, the greater the increase in HLA-DR+CD38+CD8+ T cells post-treatment, the more significant the reduction in tumor size was observed [104]. In contrast, among melanoma patients (*n* = 29) receiving various aPD-1 combination therapies, the levels of HVEM+CD8+ T and CD69+CD8+ T cells at three months post-treatment were significantly negatively correlated with PFS [105].

Among the 22 included studies, only the work by Capone et al. prospectively validates the association between CD73^+^ PD-1^+^ CD8^+^ T cells and nivolumab efficacy, providing Level 2A evidence [89]. In contrast, the study by Geng et al., based on a retrospective cohort with fewer than 100 patients, yields Level 4B evidence [103]. All other studies are rated as Level 4A. This indicates that researchers have begun to intentionally explore the functional roles of CD8^+^ T cells in prospective settings; however, the current investigations remain limited to small exploratory cohorts and can only generate preliminary hypotheses.

Given that effective CD8+ T cell activation is central to all therapeutic modalities, from monotherapy to combination regimens, the field will develop unified and more predictive surface markers. These markers will subsequently be validated in multicenter prospective studies to pave their way for clinical application.

#### CD4+T

Similar to CD8+ T cells, CD4+ T cells can be classified into naïve and memory CD4+ T cells based on their differentiation status, while activated effector CD4+ T cells can be further categorized into Th, Tfh, and Treg subsets based on their function [108]. Researchers have also explored whether these functional subsets of CD4+ T cells can serve as peripheral blood biomarkers for predicting ICI treatment efficacy (Table 4).

**Table 4:** CD4+T cells as predictive biomarkers

Biomarker	Cancer(n)	Treatment		Timepoint	Correlation	Association with clinical outcome		Level*	Ref
		Drug name	ICI type			Response	Survival		
CD62L^low^ CD4+T	NSCLC (*n* = 126)	nivo	aPD-1 mono	baseline	positive	DCR (*p* < 0.0001)	PFS (*p* < 0.0001)	2B	[109]
CD25+FOXP3+CD4+T	NSCLC (*n* = 126)	nivo	aPD-1 mono	baseline	negative	DCR (*p* < 0.034)		2B	[109]
CD26highCD4 +T	Melanoma (*n* = 69)	nivo	aPD-1 mono	baseline	positive		OS (*p* = 0.010) and PFS (*p* = 0.014)	2A	[110]
the occupancy rate of PD-1 on eTreg (CD4+CD45RA-Foxp3hi)	solid tumor (*n* = 32)	nivo	aPD-1 mono	post-treatment	negative	CBR (6 months and 12 months) (*p* = 0.0105, *p* = 0.0486)		4A	[111]
Helios+Treg (CD4+CD25+CD127lowFoxp3+)	RCC(*n* = 43)	nivo	aPD-1 mono	baseline	positive	CBR (6 months) (*p* < 0.01)	PFS (*p* = 0.003)	4A	[112]
CD4+T	NSCLC (*n* = 79)	camre/tisle/pembro+platinum-based chemo	aPD-1+chemo	baseline	positive	DCR (*p* < 0.05)	PFS (*p* = 0.0002) and OS (*p* < 0.001)	4A	[113]
PD-1+CD4+T	NSCLC (*n* = 27)	nivo+ carboplatin	aPD-1+chemo	baseline	positive	pCR (*p* = 0.045)		4A	[114]
PD-1+CD4+T	GC(*n* = 169)	sinti+ oxaliplatin + capecitabine	aPD-1+chemo	baseline	positive	CBR (6 months) (*p* < 0.001)	PFS (*p* < 0.001) and OS (*p* < 0.001)	3B	[115]
Th1-like CD4+T (T-bet+CX3CR1+KLRG1+CD57+CD4+T)	HCC(*n* = 13)	TACE +pembro	aPD-1+chemo	baseline	positive	ORR (*p* < 0.05)		4A	[116]
CD4+TCM(TCF-1+CCR7+CD4+T)	HCC(*n* = 13)	TACE +pembro	aPD-1+chemo	baseline	negative	ORR (*p* < 0.01)		4A	[116]
CD4+TCM (CD3+ CD62L+ CD45RA- CD4+T)	SCLC (*n* = 32)	atezo+ carboplatin + etoposide	aPD-L1+chemo	dynamic change	negative		OS (*p* = 0.044)	4A	[95]
Th17(CD3+CD4+CXCR3–CCR4+CCR5- CCR6+T)	SCLC (*n* = 32)	atezo+ carboplatin + etoposide	aPD-L1+chemo	dynamic change	negative		OS (*p* = 0.006)	4A	[95]
Treg (CD3+CD4+CD45RA+FOXP3+)	EAC(*n* = 24)	neo atezo+CRT	aPD-L1+CRT	baseline	negative	pCR (*p* = 0.02)	PFS (*p* = 0.04)	4A	[117]
Ki67+ Treg (CD3+CD4+CD127loCD25+/high)	cervical cancer (*n* = 16)	nivo+CRT	aPD-1+CRT	baseline	negative	DCR (*p* = 0.0328)		4A	[118]
LAG3+ PD-1+Treg	HCC(*n* = 29)	atezo+bevacizumab	aPD-L1+target	baseline	negative		OS (*p* = 0.044)	4A	[97]
CD4+T and TIM-3+CD4+T	ACC (*n* = 21)	camre + apatinib	aPD-1+target	baseline	negative	ORR (*p* = 0.055，*P* = 0.03)		4A	[96]

Biomarker: *CD4*+*TCM* central memory CD4+ T cell

Cancer: *NSCLC* non-small-cell lung cancer, *RCC* renal cell carcinoma, *GC* gastric cancer, *HCC* hepatocellular carcinoma, *SCLC* small cell lung cancer, *EAC* esophageal adenocarcinoma, ACC adrenal cortical carcinoma

Treatment: *TACE* transarterial chemoembolization, *CRT* chemoradiation therapy

Clinical outcome: *DCR* disease control rate, *PFS* progression-free survival, *OS* overall survival, *CBR* clinical benefit rate, *pCR* pathological complete response, *ORR* objective response rate

* Evidence levels: defined as in Table 1

##### APD-1/aPD-L1 monotherapy

In studies focusing on the application of aPD-1/aPD-L1 monotherapy in patient, one study involving 126 NSCLC patients treated with nivolumab demonstrated that PR/SD patients had significantly higher baseline levels of CD62L^low^ CD4+ T cells (CD62L^low^ indicates activation by antigen stimulation) and significantly lower levels of Treg (CD25+FOXP3+CD4+ T) cells, while patients with PFS > 500 days also exhibited a higher baseline percentage of CD62L^low^ CD4+ T cells [109]. Some studies have explored the specific functional subsets after activation. Galati et al. identified a population of CD26highCD4+ T cells in melanoma patients (*n* = 69) treated with nivolumab (this subset can simultaneously secrete Th17 effector molecules such as IL-17A, IL-22, and Th1 effector molecule IFN-γ, exhibiting multifunctionality and cytotoxicity [119]). A high baseline percentage ( > 7.3%) of this cell population was significantly associated with longer OS and PFS [110]. Regarding Treg cells, in addition to focusing on the level of the cells themselves, a study in solid tumor patients (*n* = 32) treated with nivolumab also examined the PD-1 occupancy rate on eTreg post-treatment [111]. They found that patients who still had clinical benefits (CR, PR, SD) at 6 months and 12 months showed significantly lower PD-1 occupancy rates on eTreg compared to those without clinical benefits [111]. However, the results of the REVOLUTION trial (*n* = 43) indicated that metastatic RCC patients responding to nivolumab (CR, PR or SD ≥ 6 months) exhibited higher baseline levels of Helios+ Treg, and patients with baseline Helios+ Treg > 34.3% demonstrated longer PFS [112]. This study suggests that there may be certain heterogeneity within Treg cells, warranting further exploration for the precise identification of Treg cell markers.

##### The combination of aPD-1/aPD-L1 and other treatments

In studies investigating the combination of aPD-1/aPD-L1 with chemotherapy, results have shown that the total CD4+ T cell level also has certain predictive potential. Yang et al. reported that in NSCLC patients receiving various PD-1 inhibitors combined with platinum-based chemotherapy (*n* = 79), patients achieving CR/PR/SD exhibited significantly higher baseline proportions of CD4+ T cells, and high levels of CD4+ T cells before and after treatment were positively correlated with OS and PFS [113]. PD-1+CD4+ T cells, representing broadly activated CD4+ T cells, also showed significant positive correlations with pCR in NSCLC patients (*n* = 27) [114] and with durable clinical benefit, PFS, and OS in GC patients (*n* = 169) [115], all of whom received aPD-1 therapy combined with chemotherapy. Regarding specific functional subsets, Pinato et al. found in 13 HCC patients treated with TACE combined with pembrolizumab that responders (CR/PR) had a cluster of Th1-like CD4+ T cells characterized by high expression of T-bet, CX3CR1, KLRG1, and CD57 at baseline, whereas non-responders showed enrichment of central memory CD4+ T cell (TCF-1+CCR7+CD4+ T) at baseline [116]. Additionally, Schmälter et al. observed in 32 SCLC patients receiving atezolizumab combined with chemotherapy that patients with reduced central memory CD4+ T cells (CD62L+ CD45RA- CD4+ T) or Th17 subsets (CD4+CXCR3–CCR4+CCR5- CCR6+ T) after two treatment cycles had longer cumulative survival [95].

What’s more, Treg also exhibited a negative correlation with efficacy in the research on the combination of aPD-1/aPD-L1 with chemoradiotherapy or targeted therapy. Van den Ende et al. reported that in patients with esophageal adenocarcinoma (*n* = 24) who received neoadjuvant atezolizumab combined with chemoradiation therapy (CRT), the non-pCR group had a higher baseline percentage of Tregs, and a high percentage of Tregs was associated with poorer PFS [117]. Similarly, in patients with locally advanced cervical cancer (*n* = 16) treated with nivolumab combined with CRT, Ki67+ Tregs were also highly expressed in PD patients [118]. Additionally, Lee et al. found in HCC patients (*n* = 29) receiving atezolizumab combined with bevacizumab that patients with LAG3% > 9.08% in PD-1+ Tregs exhibited reduced OS [97]. Beyond Tregs, CD4+ T cells expressing the exhaustion marker TIM-3 also showed a negative correlation with therapeutic response in adrenocortical carcinoma patients (*n* = 21) treated with camrelizumab combined with apatinib [96].

A total of 13 studies investigating CD4^+^ T cell-related biomarkers are included. What is worthy of attention is that one study prospectively validated CD26highCD4^+^ T cells as a predictive biomarker for nivolumab efficacy, providing Level 2A evidence [110], and another subgroup analysis of a prospective cohort demonstrated the predictive value of CD62L^l o w^ CD4^+^ T cells and CD25^+^ FOXP3^+^ CD4^+^ T cells, corresponding to Level 2B evidence [109]. What’s more, the retrospective cohort study conducted by Gao et al. provides Level 3B evidence supporting the association between PD-1^+^ CD4^+^ T cells and treatment efficacy [115]. Apart from the above three studies, all the remaining studies have been rated as 4A-level evidence. This distribution of evidence levels closely resembles that observed for CD8^+^ T cells, suggesting that studies on CD4^+^ T cells likewise remain in the exploratory phase based on small-scale prospective cohorts.

#### Other types of T cells

##### Mucosal-associated invariant T cells (MAIT cell, table 5)

MAITs are a group of atypical T cells with a semi-invariant TCR composed of the Vα7.2-Jα33 chain, which recognizes antigens presented by MHC class I-related protein 1 [120]. MAIT cells can secrete cytotoxic effector molecules in a TCR-independent manner and also secrete Th1- and Th17-related cytokines in a TCR-dependent manner [121], and there is no unified conclusion regarding their effect on tumors. Currently, there are several studies on peripheral blood MAIT cells as biomarkers for the efficacy of aPD-1/aPD-L1 treatment (Table 5). Biasi et al. reported that patients with melanoma (*n* = 20) who responded to nivolumab or pembrolizumab treatment (CR/PR/SD > 6 months) had a higher proportion of MAIT cells at baseline and after the first treatment cycle [122]. Meanwhile, Shi et al. collected a total of 105 peripheral blood samples from NSCLC patients treated with various aPD-1-based therapies before (*n* = 35) and after (*n* = 70) treatment for analysis [123]. They found that CR and PR patients had higher levels of GZMB+ MAIT and IFN-γ+ MAIT (anti-tumor MAIT) after treatment compared to SD patients, and CR and PR patients showed a reduction in IL-17A+ MAIT (pro-tumor MAIT) levels post-treatment, while PD and SD patients did not exhibit this difference [123]. Additionally, in studies related to aPD-1 combined with chemotherapy, Qu et al. performed scRNA-seq on baseline peripheral blood from 6 NSCLC patients and identified a migratory subset of MAIT cells (CXCR6+CD8+MAIT) [124]. Flow cytometry results (*n* = 30) showed that patients with a baseline CXCR6+CD8+MAIT/CD8+MAIT ratio ≥ 35.9% exhibited an improvement in PFS compared to those with a ratio < 35.9% [124]. Based on the above research findings, MAIT cells exhibiting positive effects predominantly express cytotoxicity and migratory markers, whereas IL-17A+ MAIT cells with negative effects display phenotypic similarities to Th17 cells. These observations suggest that the precise phenotypic characterization of MAIT cells partially overlaps with established findings in CD8+ T cells and CD4+ T cells. Accordingly, future studies may leverage existing knowledge from CD4+ T cell and CD8+ T cell research to more accurately define the phenotypic and functional properties of MAIT cells.

**Table 5:** Other types of T cells and TCR indicators as predictive biomarkers

Biomarker	Cancer(n)	Treatment		Timepoint	Correlation	Association with clinical outcome		Level*	Ref
		Drug name	ICI type			Response	Survival		
MAIT	Melanoma (*n* = 20)	nivo/pembro	aPD-1 mono	baseline	positive	CBR (6 months) (*p* = 0.023)		4A	[122]
GZMB+MAIT and IFN-γ+MAIT	NSCLC (*n* = 82/53)	none	aPD-1 mono	post-treatment	positive	ORR (*p* = 0.006, *p* = 0.04)		4B	[123]
IL-17A+ MAIT	NSCLC (*n* = 79)	none	aPD-1 mono	dynamic change	negative	ORR (*p* = 0.028)		4B	[123]
CXCR6+CD8+MAIT	NSCLC(*n* = 30)	pembro+chemo	aPD-1+chemo	baseline	positive		PFS (*p* = 0.0008)	4A	[124]
NKT and CD3brightNKT	Melanoma (*n* = 17)	nivo/pembro	aPD-1 mono	dynamic change	positive	CBR (6 months) (*p* = 0.01, *p* = 0.007)		4A	[125]
NKT	solid tumor (*n* = 89)	nivo/pembro/durva/avelumab/atezo	aPD-1/aPD-L1 mono	baseline	positive		PFS (*p* = 0.0078) and OS (*p* = 0.0075)	4A	[126]
NKT	solid tumor (*n* = 64)	nivo/pembro/durva/avelumab/atezo	aPD-1/aPD-L1 mono	post-treatment	positive		PFS (*p* = 0.0027) and OS (*p* = 0.0037)	4A	[126]
NKT	NSCLC (*n* = 79)	camre/tisle/pembro+platinum-based chemo	aPD-1+chemo	post-treatment	positive		OS (*p* = 0.035)	4A	[113]
TRGC2+NKT	ESCC(*n* = 12)	neo pembro+ paclitaxel + cisplatin	aPD-1+chemo	post-treatment	positive	MPR (*p* = 0.014)		4A	[127]
TCR clonotype count	pleural mesothelioma (*n* = 49)	nivo mono/+ipi	aPD-1 mono/+aCTLA-4	baseline	positive		OS (*p* = 0.033)	4A	[128]
expanded TCR clonotype count	SCBC (*n* = 7) and NEPC (*n* = 8)	pembro+ (etoposide +cisplatin/carboplatin)/pembro+ docetaxel+carboplatin	aPD-1+chemo	dynamic change	positive		PFS (*p* = 0.09，*P* = 0.011)	4A	[129]
TCR diversity	Melanoma (*n* = 25)	camre+ apatinib	aPD-1+target	baseline	positive		PFS (*p* = 0.059) and OS (*p* = 0.025)	4A	[130]
TCR diversity	HNSCC (*n* = 39)	nivo+cetuximab	aPD-1+target	baseline	positive		OS (*p* = 0.097)	4A	[131]
TCR diversity	RCC(*n* = 25)	nivo	aPD-1 mono	dynamic change	negative		PFS (*p* = 0.0011) and OS (*p* = 0.0082)	4A	[132]
TCR clonality	Melanoma (*n* = 38)	nivo/pembro	aPD-1 mono	baseline	positive	ORR (*p* < 0.0016)	PFS (*p* = 0.034)	4B	[133]
TCR clonality	MPLC(*n* = 36)	sinti	aPD-1 mono	baseline	positive	ORR (*p* < 0.01)		4A	[84]
TCR clonality	HER2-negative breast cancer (*n* = 43)	pembro	aPD-1 mono	baseline	positive		PFS (*p* = 0.04)	4A	[134]
TCR clonality	solid tumor(*n* = 24)	(pembro/nivo/atezo/durva/sinti) mono/+chemo/target	aPD-1/aPD-L1 mono/combi	post-treatment	negative	CBR (6 months) (*p* = 0.00021)	PFS (*p* = 0.098) and OS (*p* = 0.068)	4A	[135]
TCR convergence	NSCLC (*n* = 13)	nivo+ platinum-based chemo/nivo+ipi	aPD-1+chemo/aPD-1+aCTLA-4	baseline	positive	MPR (*p* = 0.036)	DFS (*p* = 0.0067)	4A	[136]

Biomarker: *MAIT* mucosal-associated invariant T cell, *NKT* natural killer T cell, *TCR* T cell receptor

Cancer: *NSCLC* non-small-cell lung cancer, *ESCC* esophageal squamous cell carcinoma, *SCBC* small cell bladder cancer, *NEPC* neuroendocrine prostate cancer, *HNSCC* head and neck squamous cell carcinoma, *RCC* renal cell carcinoma, *MPLC* multiple primary lung cancer

Clinical outcome: *CBR* clinical benefit rate, *ORR* objective response rate, *PFS* progression-free survival, *OS* overall survival, *MPR* major pathological response

* Evidence levels: defined as in Table 1

##### Natural killer T cells (NKT cell, table 5)

NKT cells, as unconventional T cells possessing characteristics of both NK cells and T cells, recognize glycolipid antigens presented by CD1d molecules and rapidly generate potent cytokine responses [137], generally positively correlating with therapeutic efficacy (Table 5). Kasanen et al. observed a significant increase in NKT and CD3brightNKT cells only in melanoma patients (*n* = 17) responding to aPD-1 monotherapy (CR/PR/SD > 6 months) [125]. Similarly, Zhou et al. found that high levels of NKT cells at baseline and after one cycle of treatment could predict better PFS and OS in solid tumor patients (*n* = 89 and 64) receiving various aPD-1/aPD-L1 monotherapies [126]. In studies related to aPD-1 combined with chemotherapy, Yang et al. also reported a significant positive correlation between post-treatment NKT levels and OS in NSCLC patients (*n* = 79) [113]. Furthermore, Shang et al. found that only patients with MPR had a significant increase in the peripheral blood TRGC2+NKT cell subset in locally advanced resectable ESCC patients (*n* = 12) treated with neoadjuvant pembrolizumab combined with chemotherapy [127].

In contrast to the extensive research on CD8^+^ and CD4^+^ T cells, all studies on MAIT and NKT cells are exclusively Level 4A or 4B, underscoring their nascent stage of investigation.

### TCR metrics

The interaction between TCR and peptide-MHC-complex serves as the antigen recognition signal for T cells, which is crucial for T cell activation [82]. Meanwhile, the TCR sequence acts as a unique identifier for T cells, and the recognition of specific sequences enables the continuous tracking of specific TCR clones [138]. To broadly recognize antigens, the body generates a highly diverse TCR repertoire through V(D)J recombination, and TCR clones that successfully recognize antigens undergo reactive expansion. Therefore, various characteristics of TCRs, such as diversity and clonality, hold significant potential for predicting the efficacy of ICIs [108, 139] (Table 5).

Some studies have investigated the correlation between the simplest TCR clonotype count and the efficacy of aPD-1/aPD-L1 treatment. Desai et al. collected peripheral blood mononuclear cell (PBMC) samples from patients before (*n* = 49) and after treatment (*n* = 39) in the NivoMes trial (*n* = 34, Phase II, nivolumab monotherapy) and the INITIATE trial (*n* = 34, Phase II, nivolumab combined with ipilimumab), and found that patients with a high baseline TCR clonotype count exhibited significantly longer OS [128]. Gu et al. focused more specifically on TCR clonotypes accounting for more than 0.1% of total PBMC cells and discovered that the fold expansion of these clonotypes at cycles 7 and 17 compared to baseline positively correlated with PFS in patients with small cell bladder cancer (*n* = 7) and neuroendocrine prostate cancer (*n* = 8) treated with pembrolizumab combined with chemotherapy [129].

However, the TCR clonotype count can only represent the total number but cannot reflect the relative size of each clonotype, therefore, TCR diversity is a relatively more comprehensive metric. TCR diversity includes richness and evenness, where the former measures the number of different specific TCR clonotypes, and the latter measures the relative abundance of different specific TCR clonotypes [140, 141]. In two studies utilizing aPD-1 therapy combined with angiogenesis inhibitors, melanoma patients (*n* = 25) with high baseline TCR diversity showed significantly longer OS [130]. In patients with HNSCC (*n* = 39), while TCR diversity demonstrated the same trend with OS, the association did not reach statistical significance *(p* = 0.097) [131]. But notably, when only HPV-negative HNSCC patients were analyzed, the association became significant (*p* = 0.048) [131]. Regarding post-treatment TCR diversity, a study involving 25 renal cancer patients treated with nivolumab showed that patients with a decreased diversity index after the first dose had significantly longer PFS and OS compared to those without a decrease [132], suggesting that higher TCR diversity post-treatment may be unfavorable for treatment efficacy.

TCR clonality is another frequently used metric, and clonality represents antigen-driven T-cell expansion, thus to some extent, it is also a marker of tumor reactivity [83]. Hogan et al. calculated the peripheral blood TCR DE_50_ score (a low DE_50_ score indicates high clonality) in melanoma patients (*n* = 38) treated with nivolumab or pembrolizumab and found that a baseline DE_50_ < 20.4% was associated with a favorable response (CR/PR) at 12 weeks and longer PFS [133]. Positive correlations between baseline TCR clonality and response as well as PFS were also observed in multiple primary lung cancer patients (*n* = 36) [84] and HER2-negative breast cancer patients (*n* = 43) [134] treated with aPD-1 monotherapy. Regarding TCR clonality after treatment, Li et al. found that in solid tumor patients (*n* = 24) receiving various treatments based on aPD-1/aPD-L1, patients with PR/SD > 6 months had significantly lower TCR clonality compared to other patients, and patients with lower clonality also exhibited better PFS and OS than those with higher clonality [135], indicating that post-treatment clonality may be negatively correlated with efficacy. However, due to the limited literature, no unified conclusion could be drawn.

Additionally, some new indicators are gradually being applied. During gene rearrangement, due to the degeneracy of the DNA sequence, multiple codons can encode the same amino acid, and different V(D)J rearrangements can ultimately produce identical TCR amino acid sequences. This phenomenon is called TCR convergence, which represents the antigen specificity of TCR [142]. Liu et al. found that in NSCLC patients treated with neoadjuvant nivolumab combined with chemotherapy (*n* = 13) or nivolumab combined with ipilimumab (*n* = 4), TCR sequencing was performed on the baseline peripheral blood of 13 patients. The baseline peripheral TCR convergence in the MPR group was significantly higher than in the non-MPR group, and patients with high baseline convergence had longer DFS than those with low convergence [136].

Various TCR features hold predictive value for ICI efficacy: at baseline, higher clonotype count, diversity, clonality, and convergence are positively correlated with better treatment outcomes; whereas post-treatment, increased diversity or elevated clonality may be negatively associated with therapeutic efficacy. All ten studies on TCR-related biomarkers are small-scale exploratory analyses, including one Level 4B retrospective study and nine Level 4A prospective studies. This indicates that research on TCR biomarkers remains at a very early exploratory stage, which may be attributed to the complexity and high cost of their detection processes.

### B cells

Studies exploring peripheral blood B cells as predictive biomarkers of efficacy are still mainly focused on research employing aPD-1/aPD-L1 monotherapy (Table 6). Xia et al. evaluated the correlation between baseline levels of circulating B cells and efficacy in NSCLC patients (*n* = 120) receiving nivolumab treatment, and found that responding patients had higher baseline levels of CD19+B cells and IgM+B cells, and patients with higher percentages of these two cell populations both exhibited higher PFS [143]. Similarly, a positive correlation between baseline B cells and best overall response (BOR) was also observed in NSCLC (*n* = 19) and mesothelioma (*n* = 16) patients, but in this study, CD27- IgD- B cells (double-negative B cells, immunosuppressive) were negatively correlated with BOR [144]. Based on CD27 and IgD expression, a subset of unswitched memory B cells (CD27+IgD+) can be identified in peripheral blood. In a study involving 60 HNSCC patients treated with nivolumab, patients with CR/PR/SD > 6 months had a lower percentage of unswitched B cells (CD21+CD27+IgM+IgD+) and a higher percentage of plasmablasts (CD38++IgM-) at baseline [145]. Conversely, in Carril-Ajuria’s study, unswitched B cells (CD19+CD27+IgM+IgD+) were enriched at baseline in clear cell renal carcinoma patients (*n* = 44) who achieved CR/PR after receiving nivolumab and were associated with improved OS and PFS [146], which represents an opposite conclusion to the previous study.

**Table 6:** B cells, NK cells and myeloid cells as predictive biomarkers

Biomarker	Cancer(n)	Treatment		Timepoint	Correlation	Association with clinical outcome		Level*	Ref
		Drug name	ICI type			Response	Survival		
CD19+B cell and IgM+B cell	NSCLC (*n* = 120)	nivo	aPD-1 mono	baseline	positive	CBR (6 months) (*p* = 0.004，*P* < 0.001)	PFS (*p* = 0.002，*P* = 0.004)	2B	[143]
B cell	NSCLC (*n* = 19) and mesothelioma (*n* = 16)	nivo/pembro	aPD-1 mono	baseline	positive	ORR (*p* < 0.01)		4B	[144]
CD27- IgD- B cell	NSCLC (*n* = 19) and mesothelioma (*n* = 16)	nivo/pembro	aPD-1 mono	baseline	negative	ORR (*p* < 0.05)		4B	[144]
unswitched B cell (CD21+CD27+IgM+IgD+)	HNSCC (*n* = 60)	nivo	aPD-1 mono	baseline	negative	CBR (6 months) (*p* = 0.017716)		4A	[145]
plasmablast (CD38++IgM-)	HNSCC (*n* = 60)	nivo	aPD-1 mono	baseline	positive	CBR (6 months) (*p* = 0.023564)		4A	[145]
unswitched B cell (CD19+CD27+IgM+IgD+)	ccRCC(*n* = 44)	nivo	aPD-1 mono	baseline	positive	ORR (*p* = 0.006)	PFS (*p* = 0.048) and OS (*p* = 0.002)	4A	[146]
NK	NPC (*n* = 88)	KL-A167	aPD-L1 mono	baseline	negative		PFS (*p* < 0.001) and OS (*p* < 0.001)	4A	[147]
NK	NSCLC (*n* = 54)	nivo	aPD-1 mono	baseline	negative		OS (*p* = 0.002)	4A	[90]
NK	NSCLC (*n* = 30)	pembro/nivo/durva/atezo	aPD-1/aPD-L1 mono	baseline	positive	CBR (6 months) (*p* < 0.05)	PFS(*p* = 0.005)	4B	[148]
CD56^dim^CD16+ NK	NSCLC (*n* = 30)	pembro/nivo/durva/atezo	aPD-1/aPD-L1 mono	baseline	positive	CBR (6 months) (*p* < 0.05)		4B	[148]
CD56^dim^CD16+NK	NSCLC (*n* = 57)	nivo/pembro	aPD-1 mono	baseline	positive		PFS (*p* = 0.006)	4A	[149]
PD-1+CD56^bright^CD16- NK	NSCLC (*n* = 55)	pembro/nivo/durva/atezo	aPD-1/aPD-L1 mono	baseline	negative		OS (*p* = 0.047)	4A	[150]
KIR2DL2/DL3+NK	RCC(*n* = 49)	nivo	aPD-1 mono	baseline	positive	CBR (6 months) months (*p* < 0.001)	OS (*p* = 0.036)	4A	[112]
KIR2DL2/DL3+NK	RCC(*n* = 52)	nivo	aPD-1 mono	post-treatment	positive	CBR (6 months) (*p* < 0.05)	PFS (*p* = 0.0002) and OS (*p* < 0.0001)	4A	[112]
classical CD14+CD16-monocyte	Melanoma (*n* = 42)	pembro/nivo	aPD-1 mono	baseline	negative	DCR (*p* = 0.034)		4B	[151]
classical CD14+CD16-monocytee	primary liver cancer (*n* = 23)	camre/sinti mono/+Lenvatinib	aPD-1 mono/+target	baseline	positive	ORR (*p* = 0.022)		4A	[152]
non-classical CD14dimCD16+monocyte	NSCLC (*n* = 46)	nivo/pembro	aPD-1 mono	post-treatment	positive		OS(*p* = 0.004)	4A	[149]
intermediate CD14+CD16+monocyte and eMDSC(CD14-HLA-DR-CD33+CD11b+)	EAC(*n* = 24)	neo atezo+CRT	aPD-L1+CRT	baseline	negative	pCR (*p* = 0.01, *p* = 0.04)		4A	[117]
slan + monocyte	NSCLC (*n* = 30)	pembro/nivo/durva/atezo	aPD-1/aPD-L1 mono	baseline	positive	CBR (6 months) (*p* < 0.01)	PFS (*p* = 0.0006)	4B	[148]
PMN-MDSC(CD33dimHLA-DRlow/-CD14–CD66b+Lin-) and M-MDSC(CD33+HLA-DRlow/-CD14+CD66b-)	Melanoma (*n* = 19)	nivo/pembro/nivo mono/+ipi	aPD-1 mono/+aCTLA-4	post-treatment	negative	DCR (*p* < 0.01，*P* < 0.05)		4A	[153]
PD-L1+, B7H3+ and CD115+ myeloid cell	GC(*n* = 96)	nivo	aPD-1 mono	baseline	negative		PFS (*p* = 0.019，*P* < 0.001，*P* = 0.012) and OS(*p* = 0.015，*P* = 0.032，*P* = 0.018)	4A	[154]
LAG3+,CD155+ and CD115+ myeloid cell	GC(*n* = 96)	nivo	aPD-1 mono	post-treatment	negative		PFS (*p* = 0.004，*P* = 0.017，*P* = 0.020) and OS (*p* = 0.028，*P* = 0.032，*P* = 0.017)	4A	[154]

Biomarker: *NK* natural killer cell, *eMDSC* early myeloid-derived suppressor cell, *PMN-MDSC* polymorphonuclear myeloid-derived suppressor cell, *M-MDSC* monocytic myeloid-derived suppressor cell

Cancer: *NSCLC* non-small-cell lung cancer, HNSCC head and neck squamous cell carcinoma, *ccRCC* clear cell renal cell carcinoma, *NPC* nasopharyngeal carcinoma, *RCC* renal cell carcinoma, *HCC* hepatocellular carcinoma, *EAC* esophageal adenocarcinoma, *GC* gastric cancer

Treatment: *CRT* chemoradiation therapy

Clinical outcome: *CBR* clinical benefit rate, *ORR* objective response rate, *PFS* progression-free survival, *OS* overall survival, *DCR* disease control rate, *pCR* pathological complete response

* Evidence levels: defined as in Table 1

The role of B cells in antitumor immunity remains a relatively underexplored area, and consequently, the investigation of circulating B cells as biomarkers is correspondingly limited. Presently, only CD19^+^ and IgM^+^ B cells have garnered Level 3A evidence from large retrospective cohorts [143], while the majority of other proposed biomarkers remain at exploratory stages (Levels 4A-4B), lacking strong prospective validation.

### NK cell

NK cells can serve as a biomarker for the efficacy of aPD-1/aPD-L1 therapy, yet they exhibit contradictions across different studies (Table 6). In the post hoc analysis of Phase II trial KL167-2–05-CTP, including 88 nasopharyngeal carcinoma patients who were treated with KL-A167 (a PD-L1 inhibitor) and had baseline NK cell data, researchers found that patients with lower NK cell percentages showed significantly longer PFS and OS compared to those with higher NK cell percentages [147]. A negative correlation between NK cell levels and OS was also observed in NSCLC patients (*n* = 54) treated with nivolumab [90]. However, in another study involving 30 NSCLC patients treated with various aPD-1/aPD-L1 monotherapies, patients with higher baseline NK cell counts exhibited lower disease progression rates within 6 months and significantly longer PFS [148]. These conflicting findings indicate that relying solely on the overall abundance of NK cells as a predictive biomarker has clear limitations. Therefore, achieving precise classification and identification of NK cell subtypes is critical for discovering effective predictive indicators.

Classically, some studies classified NK cells into two major subsets: CD56^dim^CD16+NK (cytotoxic NK) and CD56^bright^CD16-NK (immunoregulatory NK) [155]. In the prior study involving 30 NSCLC patients, patients with higher baseline levels of CD56^dim^CD16+NK also showed lower disease progression rates within 6 months [148]. Another study involving 57 NSCLC patients treated with nivolumab/pembrolizumab also demonstrated a positive correlation between CD56^dim^CD16+NK cells and better PFS [149]. In contrast, Gascón-Ruiz et al. exhibited a negative correlation between PD-1+CD56^bright^CD16-NK and OS in NSCLC patients (*n* = 55) treated with various aPD-1/aPD-L1 monotherapies [150]. Additionally, Santagata et al. identified a population of KIR2DL2/DL3+NK cells (capable of killing abnormal or tumor cells with downregulated or absent MHC class I molecules) in metastatic RCC patients (*n* = 57) treated with nivolumab [112]. Patients who responded to treatment exhibited higher levels of KIR2DL2/DL3+NK both at baseline and post-treatment, and patients with baseline KIR2DL2/DL3+NK > 35.3% showed significantly longer OS, while patients with post-treatment KIR2DL2/DL3+NK > 23.3% demonstrated longer PFS and OS [112].

It is evident that the predictive value of NK cell subsets highly depends on their differentiation status, exhibiting complex and dynamic characteristics. Specifically, the cytotoxic subset CD56^dim^CD16+ NK is associated with prolonged PFS in NSCLC patients [149], and KIR2DL2/DL3+ NK indicates survival benefit throughout the treatment process in kidney cancer [112], while the regulatory CD56^bright^CD16- NK is associated with poor prognosis [150]. However, evidence grading shows that all existing studies on NK cells fall within Levels 4A or 4B, indicating that research on NK cells as biomarkers for PD-1 inhibitor efficacy remains at an exploratory stage. What’s more, current research on peripheral blood NK cells predominantly focuses on the classical classification system, with relatively limited exploration of other characteristic markers.

### Myeloid cells

Several studies have explored the potential of myeloid cells as indicators of therapeutic efficacy (Table 6). Monocytes within myeloid cells can be classified into classical (CD14+CD16-), intermediate (CD14+CD16+), and non-classical (CD14+CD16++) types based on cell surface proteins. Pirozyan et al. found that in melanoma patients (*n* = 42) treated with pembrolizumab/nivolumab, the frequency of classical CD14+CD16- monocytes was higher in PD patients [151]. However, this cell subset showed opposite correlations with treatment response in primary liver cancer (*n* = 23) treated with aPD-1 monotherapy or combined with Lenvatinib [152]. Similarly, Lo Russo et al. discovered that in NSCLC patients (*n* = 46) receiving the pembrolizumab/nivolumab, non-classical CD14dimCD16+ monocytes were positively associated with longer OS [149]. As for intermediate CD14+CD16+ monocytes, it was found to have a negative correlation with pCR in cases of esophageal adenocarcinoma (*n* = 24) treated with neoadjuvant atezolizumab combined with CRT [117]. Given a more detailed identification of monocytes, Pettinella et al. identified a population of slan+ monocytes(a proinflammatory subpopulation within non-classical monocytes) in NSCLC patients (*n* = 30) treated with various aPD-1/aPD-L1 monotherapies [148], and patients without progression within 6 months had significantly higher baseline levels of slan + monocytes, and those with ≥7 cells/µL exhibited significantly longer PFS [148].

Moreover, in the previous study on esophageal adenocarcinoma, early myeloid-derived suppressor cells (eMDSC, reduced immunosuppressive efficacy) were found to be negatively correlated with pCR [117]. Similarly, in melanoma patients treated with aPD-1 monotherapy or in combination with aCTLA-4 (*n* = 19), non-responders exhibited significantly higher levels of polymorphonuclear and monocytic myeloid-derived suppressor cells (PMN-MDSC and M-MDSC, strongly immunosuppressive) than responders over three treatment cycles [153]. Additionally, Shoji et al. found in 96 advanced GC patients treated with nivolumab that baseline PD-L1+, B7H3+, and CD115+ myeloid cell subsets, as well as post-treatment LAG3+, CD155+, and CD115+ myeloid cell subsets, were associated with poorer PFS and OS [154].

In summary, myeloid cell subsets exert dynamic influences on therapeutic efficacy through inhibitory or activated phenotypes. Inhibitory subsets are generally associated with drug resistance, including MDSCs [117, 153], monocytes expressing inhibitory immune checkpoints [154] and intermediate monocytes [117]. In contrast, activated subsets with anti-tumor properties, such as non-classical monocytes [149] and slan^+^ monocytes [148], can promote treatment response. Classical CD14+CD16- monocytes, on the other hand, exhibit dual effects [151, 152]. This bidirectional regulatory mechanism highlights the critical importance of myeloid cell classification and their dynamic transitions during therapy (e.g., from inhibition to activation) in predicting clinical outcomes. It is noteworthy that the evidence distribution for myeloid cells shows a pattern similar to that of NK cells, being predominantly Level 4A with a few Level 4B studies. This indicates that the association between myeloid cells and therapeutic efficacy also remains at an early exploratory stage.

### Cytokines

Cytokines can regulate immune responses, and therefore, they are also widely regarded as peripheral blood biomarkers for predicting treatment efficacy. Currently, the most extensively studied cytokines are IL-6 and IL-8, both of which have demonstrated a negative correlation with treatment efficacy across different cancer types and treatment modalities (Table 7). Maiorano et al. found that responders (CR/PR/SD) to avelumab treatment in urothelial carcinoma patients (*n* = 28) had lower baseline levels of IL-6 and IL-8 [156]. Similarly, high baseline levels of IL-6 and IL-8 were also associated with poor response in patients with gastric adenocarcinoma (GAC) (*n* = 29) treated with nivolumab combined with anlotinib hydrochloride [27], and in patients with resectable esophageal adenocarcinoma (*n* = 24) treated with neoadjuvant chemoradiotherapy combined with atezolizumab [117]. Similarly, baseline IL-6 was negatively correlated with PFS in melanoma patients (*n* = 26) treated with nivolumab combined with ipilimumab [157]. Galsky et al. observed that patients with elevated IL-6 after treatment with nivolumab combined with chemoradiotherapy exhibited poorer PFS and OS in muscle-invasive bladder cancer patients (*n* = 74) [158]. Meanwhile, Smithy et al. also found that high baseline IL-8 levels were associated with poorer OS in patients with solid tumors treated with pembrolizumab after stereotactic body radiation therapy (SBRT) (*n* = 50) [159]. In addition, while some other cytokines have also been shown to be related to efficacy, the number of relevant studies is limited [117, 136, 156, 158, 160, 161]. See Table 7 for details.

**Table 7:** Cytokines as predictive biomarkers

Biomarker		Cancer(n)	Treatment		Timepoint	Correlation	Association with clinical outcome		Level*	Ref
			Drug name	ICI type			Response	Survival		
IL-6	UC(*n* = 28)		avelumab	aPD-L1 mono	baseline	negative	DCR (*p* = 0.034)		4A	[156]
	GAC(*n* = 29)		nivo+anlotinib hydrochloride	aPD-1+target	baseline	negative		OS (P＜0.01)	4A	[27]
	EAC(*n* = 24)		neo atezo+CRT	aPD-L1+CRT	baseline	negative	pCR (*p* = 0.03)		4A	[117]
	Melanoma(*n* = 26)		nivo+ipi	aPD-1+aCTLA-4	baseline	negative		PFS (*p* = 0.041)	4A	[157]
	MIBC (*n* = 74)		nivo+ gemcitabine+ cisplatin	aPD-1+chemo	baseline	negative		PFS (*p* = 0.0014)	4A	[158]
	MIBC (*n* = 74)		nivo+ gemcitabine+ cisplatin	aPD-1+chemo	dynamic change	negative		PFS (*p* = 0.011) and OS (*p* < 0.05)	4A	[158]
IL-8	UC(*n* = 28)		avelumab	aPD-L1mono	baseline	negative	DCR (*p* = 0.014)		4A	[156]
	GAC(*n* = 29)		nivo+anlotinib hydrochloride	aPD-1+target	baseline	negative		OS (*p* = 0.01)	4A	[27]
	EAC(*n* = 24)		neo atezo+CRT	aPD-L1+CRT	baseline	negative	pCR (*p* = 0.02)		4A	[117]
	EAC(*n* = 24)		neo atezo+CRT	aPD-L1+CRT	post-treatment		pCR (*p* = 0.03)		4A	[117]
	solid tumor (*n* = 50)		SBRT followed by pembro	aPD-1+radio	baseline	negative		OS (*p* = 0.001)	4A	[159]
CXCL12、SCF	NSCLC (*n* = 17)		none	aPD-1/aPD-1+aCTLA-4	baseline	positive		DFS (*p* = 0.014，*P* = 0.026)	4A	[136]
IL-2 、IFN-γ	UC(*n* = 28)		avelumab	aPD-L1 mono	baseline	positive	DCR (*p* = 0.049, *p* = 0.035)		4A	[156]
Ang-2	MIBC (*n* = 74)		nivo+ gemcitabine+ cisplatin	aPD-1+chemo	baseline	negative		MFS (*p* < 0.05) and OS (*p* = 0.0055)	4A	[158]
CCL5、VEGF	EAC(*n* = 24)		neo atezo+CRT	aPD-L1+CRT	post-treatment	negative	pCR (*p* = 0.04，*P* = 0.02)		4A	[117]
CSF-1	GC(*n* = 42)		avelumab+regorafenib	aPD-L1+target	post-treatment	negative		PFS (*p* < 0.001) and OS (*p* = 0.009)	4A	[160]
IL-9	MIBC (*n* = 39)		pembro+gemcitabine+cisplatin	aPD-1+chemo	dynamic change	positive	pCR (*p* = 0.047)		4A	[161]

Cancer: *UC* urothelial carcinoma, *GAC* gastric adenocarcinoma, *EAC* esophageal adenocarcinoma, *MIBC* muscle-invasive bladder cancer, *NSCLC* non-small-cell lung cancer, *GC* gastric cancer

Treatment: *CRT* chemoradiation therapy, *SBRT* stereotactic body radiation therapy

Clinical outcome: *DCR* disease control rate, *PFS* progression-free survival, *OS* overall survival, *pCR* pathological complete response, *DFS* disease-free survival, *MFS* metastasis-free survival

* Evidence levels: defined as in Table 1

Although IL-6 and IL-8 have demonstrated relatively consistent associations with treatment efficacy, all of the above studies are classified as Level 4A evidence, indicating that these findings remain at the hypothesis-generating stage and lack validation from large-scale prospective studies.

Peripheral blood immune cells and related indicators—including lymphocyte counts (e.g., ALC, NLR), T cell subsets (CD8^+^ and CD4^+^ T cells with various functional phenotypes), B cells, NK cells, myeloid cells, and cytokines (such as IL-6 and IL-8)—show promise as biomarkers for predicting response to immune checkpoint inhibitor therapy. Although current evidence for peripheral immune cell-based biomarkers remains preliminary and requires larger prospective validation to address methodological issues and variable cutoffs, they provide important complementary insights into the functional status of the host immune system and its interplay with tumor burden.

PD-1/PD-L1 inhibitors exert their effect through enhanced anti-tumor immunity, leading to reduced tumor burden. Tumor-derived and immune-related biomarkers collectively reflect this process and offer complementary predictive value, suggesting that an integrated approach could improve prognostic accuracy. Zhang et al. analyzed 202 patients from the IMpower133 trial who received atezolizumab plus etoposide–cisplatin chemotherapy and found that, when assessed using only a Cox proportional hazards model, TMB < 10 mut/Mb was not significantly associated with OS (*p* = 0.126); however, after adjustment for NLR ≥ median, TMB < 10 mut/Mb emerged as an independent adverse prognostic factor for OS (*p* = 0.001) [162]. In another retrospective cohort study including 1714 patients across 16 cancer types treated with ICIs, the probability of benefit from ICI therapy was significantly higher in the NLR low/TMB high group compared with the NLR high/TMB low group (*p* < 0.001) [163]. Similarly, in castration-resistant prostate cancer, NLR provided additional prognostic value in CTC-based stratification [164]. Multi-parameter models that integrate multiple biomarkers may offer superior prediction of immunotherapy outcomes compared to strategies relying on a single biomarker.

## Multi-parameter models

Previous sections have shown that different biomarker classes reflect distinct but complementary aspects of tumor–immune dynamics. Integrating these variables forms the conceptual and methodological foundation for multi-parameter predictive modeling

A total of 21 studies on multi-parameter predictive models are included in this analysis [126, 152, 165–183]. From 2018 to 2025, the evolution of these models exhibits two major trends. First, the parameters have progressively expanded from conventional and easily accessible variables—such as complete blood counts, serum biochemistry, and clinical characteristics—to biologically deeper features, including immune cell and cytokine profiles, tumor-related biomarkers such as ctDNA and bTMB, and more advanced markers like phosphorylation signaling and gene expression profiles in PBMCs. Second, models based on routine clinical and laboratory parameters have undergone exploratory analyses and external validation in larger cohorts, substantially improving their generalizability and predictive robustness. The following section systematically classifies and discusses these models according to the core data types on which they are based (Table 8).

**Table 8:** Multi-parameter models as predictive biomarkers

Year	Model name	ICI type		Cancer	sample size	Model parameters	Parameter type	Correlation	Performance indicators	Ref
2018	LIPI (Lung Immune Prognostic Index)		aPD-1/aPD-L1	NSCLC	training cohort: *n* = 161, validation cohort: *n* = 305	derived neutrophil-to-lymphocyte ratio and LDH	routine clinical and laboratory parameters	The good LIPI group had the best OS, PFS and DCR, but not for chemotherapy.	none	[165]
2018	iSEND (Immunotherapy, Sex, ECOG NLR & Delta NLR)		nivolumab	NSCLC	training cohort: *n* = 159, validation cohort: internal validation, bootstrap analysis	Sex, ECOG PS, NLR, and ΔNLR	routine clinical and laboratory parameters	The iSEND good patients have the best PFS rates at 3, 6, 9 and 12 months.	training cohort: 3-months AUC = 0.718, 6-months AUC = 0.740, 8-months AUC = 0.746, 12-months AUC = 0.774 validation cohort: AUC = 0.704–0.745	[166]
2020	DIREct-On (Durable Immunotherapy Response Estimation by immune profiling and ctDNA-On-treatment)		aPD-1/PD-L1 mono/+chemo/+aCTLA-4	NSCLC	training cohort: *n* = 34, validation cohort: *n* = 38	baseline plasma bTMB, peripheral CD8+T-cell levels, and early on-treatment ΔctDNA	immune cells, ctDNA and bTMB	Patients with higher DIREct-On scores had substantially longer PFS than those with lower scores	discovery cohort: accuracy = 92%, AUC = 0.93, precision = 88%, recall = 94% validation cohort: accuracy = 92%, AUC = 0.93, precision = 95%, recall = 90%	[167]
2020	a machine-learning model		nivolumab	melanoma	training cohort: *n* = 468, validation cohort: *n* = 158	16 baseline inflammatory cytokines selected via Boruta algorithm	Cytokines	Patients with low predicted clearance from the selected cytokine features have longer survival compared with those who have high clearance.	training cohort: AUC = 0.75, accuracy: 0.70, sensitivity: 0.692, specificity: 0.692, Kappa statistic: 0.385 validation cohort: AUC = 0.71	[168]
2020	partial least square discriminant analysis (PLS-DA) model		nivolumab、pembrolizumab	melanoma and NSCLC	training cohort: Mel-PD1-A(*n* = 29) validation cohort: Mel-PD1-B(*n* = 36), NSCLC-PD1(*n* = 56)	Phosphorylation signals of 88–113 peptides (after quality control)	PBMC phosphorylation signals	This model can accurately identify patients with or without clinical benefit.	training cohort: Mel-PD1-A: correct classification rate (CCR) = 93% validation cohort: Mel-PD1-B: CCR = 78%, NSCLC-PD1: CCR = 68%	[169]
2021	LIPS (Liquid immune profile-based signature)		aPD-1/aPD-L1 mono	recurrent and/or metastatic cancer	training cohort: *n* = 56, validation cohort: *n* = 33	5 peripheral blood immune cell subsets, including the absolute count of neutrophils, plasmacytoid dendritic cells, NKT cells, CD14high monocytes, PD-1+CD8+T cells	peripheral blood immune cell profiles	Patients in the low-risk group have significantly longer OS and PFS than those in the high-risk group	training cohort: C-index = 0.74, 0.5-year AUC = 0.811, 1-year AUC = 0.708, and 2-year AUC = 0.788 validation cohort: C-index = 0.71, 0.5-year AUC = 0731, 1-year AUC = 0.716, and 2-year AUC = 0.768	[126]
2021	Nomogram		aPD-(L)1 ± aCTLA-4	MSI-H mCRC	training cohort: *n* = 163, validation cohort: *n* = 146	ICI type, ECOG PS, NLR, platelet count, and prior treatment lines	routine clinical and laboratory parameters	Patients with higher scores have a lower probability of achieving 12-month PFS.	training cohort: C-index = 0.707 validation cohort: C-index = 0.641	[170]
2022	BICS (Peripheral-blood-immune-cell-based signature)		aPD-1 mono	pan-cancer	training cohort: *n* = 91, validation cohort: *n* = 29	different variations in the percentages of Treg cells, CD3+T cells, the absolute CD19+B cell and CD3+CD4+T cells counts	peripheral blood immune cell profiles	Patients in the high-BICS group had a poorer PFS and OS than those in the low-BICS group.	training cohort: 0.5-year AUC = 0.807, 1-year AUC = 0.79, and 2-year AUC = 0.774 validation cohort: 0.5-year AUC = 0.818, 1-year AUC = 0.705, and 2-year AUC = 0.707	[171]
2022	prognostic immune risk score		aPD-1	melanoma	training cohort: *n* = 29, validation cohort: *n* = 20	absolute counts of 4 immune cell subsets: CD8+CD28+ T, CD3+TCRαβ+HLA-DR+ T, CD3+TCRγδ+HLA-DR+ T, CD1c+ dendritic cell	peripheral blood immune cell profiles	Patients in the high-risk group have significantly shorter PFS than patients in the low-risk group	training cohort: 1-year AUC = 0.75 validation cohort: 1-year AUC = 0.67	[172]
2022	Nomogram Based on Monocyte-to-Lymphocyte Ratio		aPD-1/PD-L1+chemo(validation cohort chemo alone)	ESCC	training cohort: *n* = 81, validation cohort: internal validation (1000 bootstrap resamples)	monocyte-to-lymphocyte ratio, ECOG PS, and body mass index	routine clinical and laboratory parameters	Patients in the low-risk group have the best OS.	training cohort: C-index = 0.770, 12-months AUC = 0.855, 18-months AUC = 0.792, 24-months AUC = 0.744 validation cohort: consistent calibration curves for 12-, 18-, 24-month OS	[173]
2023	Cytokine-based ICI Response Index (CIRI), including preCIRI14 and edtCIRI19		aPD-1/PD-L1 mono/combi	NSCLC	training cohort: *n* = 123, validation cohort: *n* = 99	The concentrations of 14 types of cytokines at the baseline and 19 types after treatment	Cytokines	Patients in the high-risk group have a significantly worse OS than those in the low-risk group.	preCIRI14: ·training cohort: C-index = 0.667, AUC = 0.874~0.736(2-year) ·validation cohort: C-index = 0.700, AUC = 0.920~0.568(2-year) edtCIRI19: ·training cohort: C-index = 0.712, AUC ≈0.92~ 0.778(2-year) ·validation cohort: C-index = 0.751, AUC = 0.912~0.725(2-year)	[174]
2023	prognostic peripheral blood-based risk model		aPD-1/PD-L1 mono/+chemo/+aCTLA-4	pan-cancer	training cohort: *n* = 109, no validation cohort	hemoglobin, platelet count, neutrophil count, C-reactive protein, erythrocyte sedimentation rate, LDH	routine clinical and laboratory parameters	Patients in the high-risk group have worse ORR, DCR, OS and PFS in comparison to the patients in the low-risk group	none	[175]
2023	ESCLL nomogram		aPD-1	NSCLC	training cohort: *n* = 138, validation cohort: internal validation	ECOG PS, preSII (platelet count×neutrophil count/lymphocyte count), change(C-reactive protein-albumin ratio), change(lymphocyte count), postLDH	routine clinical and laboratory parameters	Patients in the high-risk group exhibited a significantly poorer PFS.	training cohort: 1-year AUC = 0.893, 2-year AUC = 0.828 validation cohort: The calibration plot revealed that the 1- and 2-year survival probabilities predicted by ESCLL nomogram had an excellent agreement with the actual observations.	[176]
2024	LORIS (Logistic Regression-based Immunotherapy-Response Score)		aPD-1/PD-L1 mono/+aCTLA-4(account for 99.8% of the total population)	pan-cancer	training cohort: *n* = 964, validation cohort: *n* = 515	TMB, history of systemic treatment, blood albumin, blood NLR, age and cancer type	routine clinical and laboratory parameters, TMB	Participants with low LORIS (binned at 0.5) have significantly worse OS and PFS compared to those with high scores.	training cohort: AUC: 0.74 (0.71, 0.77) validation cohort: AUC: 0.75 (0.70, 0.80)	[177]
2024	CABLE score (abbreviation for C-reactive protein, Albumin, Bilirubin, Lymphocytes, ECOG, EHS)		aPD-L1(atezolizumab)+bevacizumab	HCC	training cohort: *n* = 526, validation cohort: *n* = 157	C-reactive protein, albumin, bilirubin, lymphocyte count, ECOG (0/1) and extrahepatic spread (0/1)	routine clinical and laboratory parameters	Patients in the high-risk group have a significantly worse OS than those in the low-risk group.	discovery cohort: 6-months AUC = 0.774, 12-months AUC = 0.792, 18-months AUC = 0.815, C indices = 0.68–0.69 validation cohort: 6-months AUC = 0.835, 12-months AUC = 0.713, 18-months AUC = 0.754, C indices > 0.7	[178]
2024	5-gene signature model		aPD-1(pembrolizumab)+chemo	NSCLC	training cohort(PMBCR cohort): *n* = 59, validation cohort: BC cohort, *n* = 42，PBMCP cohort, *n* = 42	The expression levels of five genes: UQCRB, NDUFA3, CDKN2D, FMNL1-DT and APOL3	peripheral blood gene expression characteristics	Patients predicted as responders have significantly longer PFS compared to those predicted as non-responders.	discovery cohort: PMBCR cohort (AUC = 0.90,95% CI: 0.82–0.99) validation cohort: BC cohort (AUC = 0.89, 95% CI: 0.75–1.00), PBMCP cohort (AUC = 0.89, 95% CI: 0.80–0.99)	[179]
2024	angiogenesis-associated risk score model		aPD-1/PD-L1 mono/+aCTLA-4/+antiangiogenic therapy	esophageal cancer	training cohort: *n* = 91, no validation cohort	IL-8, angiopoietin-1 receptor, hepatocyte growth factor	Cytokines	High risk score was significantly associated with shorter OS, shorter PFS, and non-response.	training cohort: 6-month AUC = 0.863, 18-month AUC = 0.716 validation cohort: none	[180]
2024	Multimodal model for predicting response to ICIs		various aPD-1/aPD-L1-based treatments	NSCLC	training cohort: *n* = 83, validation cohort: *n* = 75	baseline normalized bTMB, early ctDNA dynamics (ΔctDNA), first RECIST response	routine clinical parameters, bTMB and ΔctDNA	Patients with higher model scores had significantly longer PFS.	training cohort: AUC = 0.878, accuracy: 0.8, sensitivity: 0.792, specificity: 0.864 validation cohort: AUC = 0.887, accuracy: 0.903, sensitivity: 0.947, specificity: 0.853	[181]
2024	PISIR (Peripheral Immune Signature of ICI-Response)		aPD-1 ± lenvatinib	primary liver cancer	training cohort: *n* = 12, testing cohort: *n* = 11, independent validation cohort: *n* = 16	classical monocyte% (CM%), B cell%, HLA-DR+ CD8+T%, NK cell CD45 expression, T cell PD-1 expression	peripheral blood immune cell profiles	The output of the PISIR model is a categorical variable, where a value of 0 represents ICI resistance and a value of 1 represents ICI responsiveness.	training cohort: AUC = 1 testing cohort: AUC = 0.95 independent validation cohort: AUC = 0.925	[152]
2025	SCORPIO (Standard Clinical and Laboratory Features for Prognostication of Immunotherapy Outcomes)		aPD-1/aPD-L1/aCTLA-4/combinations of more than one drug	pan-cancer	training cohort (MSK-I): *n* = 1628 internal test cohorts: MSK-I hold-out test set: *n* = 407, MSK-II: *n* = 2104 external validation cohorts: 10 Global Phase III Trials: *n* = 4447, MSHS Real-World Cohort: *n* = 1159	33 significantly correlated features from demographics, clinical features, complete blood count, comprehensive metabolic panel	routine clinical and laboratory parameters	OS and clinical benefit rates increase with decreasing risk. The predictive performance of this model significantly outperforms TMB and PD-L1 staining and it has specificity for ICI efficacy prediction.	Internal test cohorts: MSK-I hold-out test set: OS: median AUC(t) = 0.763 (6–30 months); Clinical benefit: AUC = 0.714 MSK-II: OS: median AUC(t) = 0.759; Clinical benefit: AUC = 0.641 External validation cohorts: 10 Global Phase III Trials: Robust performance in OS and clinical benefit prediction MSHS Real-World Cohort: OS: median AUC(t) = 0.725.	[182]
2025	MPTC model		aPD-1 mono/+chemo	HNSCC	training cohort: *n* = 56, validation cohort: internal validation, bootstrap analysis (1000 resamples)	treatment, CPS, monocyte-to-lymphocyte ratio, and platelet-to-lymphocyte ratio	routine clinical and laboratory parameters	Patients with high model scores had significantly longer PFS than those with low scores.	training cohort: AUC = 0.877, sensitivity = 0.81, specificity = 0.85 validation cohort: C-index = 0.835	[183]

Cancer: *NSCLC* non-small cell lung cancer, *MSI-H mCRC* microsatellite instability-high metastatic colorectal cancer, *ESCC* esophageal squamous cell carcinoma, *HCC* hepatocellular carcinoma, *HNSCC* head and neck squamous cell carcinom

Model parameters: *LDH* lactate dehydrogenase, *ECOG PS* Eastern Cooperative Oncology Group Performance Status, *NLR* neutrophil-to-lymphocyte ratio, *bTMB* blood tumor mutation burden, *ctDNA* circulating tumor *DNA*, *NKT* Natural killer T cell, *ICI* immune checkpoint inhibitor, Treg regulatory T cell, *SII* systemic immune inflammation index, *TMB* tumor mutation burden, RECIST response evaluation criteria in solid tumors, *CPS* combined positive score

Clinical outcome: *OS* overall survival, *PFS* progression-free survival, *DCR* disease control rate, *ORR* objective response rate

Performance indicators: *AUC* area under curve, *CCR* correct classification rate, *C-index* concordance index

### Models based on routine clinical and laboratory parameters

These models demonstrate the highest clinical feasibility and cost-effectiveness, as their parameters are routinely available in clinical practice, inexpensive, and highly standardized. This category comprises nine studies, representing the largest proportion among all included models. Early models were built on simple combinations of inflammatory markers. For example, the LIPI model, derived from a 161-patient training cohort and a 305-patient validation cohort, stratified patients using only derived neutrophil-to-lymphocyte ratio (dNLR) > 3 and LDH > ULN into three groups (good: 0 factors; intermediate: 1 factor; poor: 2 factors) [165]. The good group showed the most favorable OS, PFS, and DCR, although this pattern did not extend to chemotherapy [165]. In the same year, the iSEND model incorporated sex, Eastern Cooperative Oncology Group Performance Status (ECOG PS), NLR, and ΔNLR as parameters and demonstrated clear PFS discrimination in 159 nivolumab-treated patients with NSCLC, but remained limited to internal validation [166].

Subsequent models expanded and integrated more diverse clinical and laboratory parameters. Pietrantonio et al. developed a nomogram based on ICI type, ECOG PS, NLR, platelet count, and prior treatment lines to predict 12-month PFS in MSI-H metastatic CRC treated with aPD-(L)1 mono or in combination with aCTLA-4 [170]. Ma et al. constructed a nomogram combining monocyte-to-lymphocyte ratio, ECOG PS, and body mass index to estimate OS in ESCC patients receiving PD-1/PD-L1 inhibitors plus chemotherapy [173]. Tiainen et al. further incorporated hemoglobin, platelet and neutrophil counts, C-reactive protein, erythrocyte sedimentation rate, and LDH to develop a practical peripheral blood–based prognostic model applicable across cancer types and ICI regimens, showing consistently worse ORR, DCR, OS, and PFS in the high-risk group [175]. Similarly, the ESCLL nomogram [176], CABLE score [178], and MPTC model [183], established in NSCLC, HCC, and HNSCC respectively, demonstrated significant high-risk/low-risk stratification results, underscoring the broad utility of routine clinical and laboratory data in predicting ICI outcomes.

Building on these foundations, model development has advanced toward large-scale, multicenter, and pan-cancer integration. The SCORPIO model represents a key milestone in this evolution [182]. Trained and validated on 9,745 patients across 21 cancer types using both real-world and clinical-trial datasets, SCORPIO employed machine learning to incorporate 33 significantly correlated features spanning demographics, clinical characteristics, complete blood counts, and metabolic panels. It showed progressively higher clinical benefit and improved OS with decreasing risk, and its predictive performance markedly outperformed TMB and PD-L1.

These models generally exhibit strong predictive performance in training cohorts, with most area under curve (AUC) values exceeding 0.7 and some surpassing 0.8. However, only four studies performed external validation [165, 170, 178, 182] and one of them demonstrated decreased AUC value in validation cohorts [170], suggesting that their generalizability remains somewhat limited. Notably, the SCORPIO model maintained a stable OS predictive performance with an AUC > 0.7 even across highly heterogeneous, large-scale datasets [182]. This robustness underscores its strong translational potential and positions it as one of the most promising candidates for near-term clinical implementation.

### Models based on peripheral blood immune cell profiles

These models utilize techniques such as flow cytometry to comprehensively characterize the dynamic changes of peripheral immune cell subsets, providing more detailed immunological insights than conventional hematologic markers like NLR.

Zhou et al. developed the LIPS model in patients with recurrent or metastatic cancer receiving aPD-1/aPD-L1 monotherapy, using absolute count of neutrophils, plasmacytoid dendritic cells, NKT cells, CD14high monocytes and PD-1+CD8+T cells [126]. The model successfully distinguished high- and low-risk groups with markedly different OS in both the training cohort (*n* = 56) and the validation cohort (*n* = 33) [126]. Furthermore, Wei et al. incorporated dynamic immune-cell changes and constructed the BICS model based on variations in Treg cells, CD3^+^ T cells, and absolute counts of CD19+B cells and CD3+CD4+T cells (training *n* = 91; validation *n* = 29) [171]. Consistently, patients classified as high-risk showed significantly shorter PFS and OS across multiple tumor types treated with aPD-1 [171]. In a more refined immune-profiling strategy, Chi et al. screened 66 immune-cell subpopulations and identified four predictive subsets (CD8^+^ CD28^+^ T cells, CD3^+^ TCRαβ^+^ HLA-DR^+^ T cells, CD3^+^ TCRγδ^+^ HLA-DR^+^ T cells, and CD1c^+^ dendritic cells) to construct an immune risk score that effectively predicted PFS in melanoma patients treated with aPD-1 monotherapy [172]. Further extending this framework, Xue et al. integrated parameters of classical monocytes%, B cells%, HLA-DR^+^ CD8^+^ T cells%, NK cell CD45 expression, T cell PD-1 expression to establish the PISIR model in primary liver cancer [152]. Notably, the model outputs were directly translated into a binary classification of “ICI-sensitive” versus “ICI-resistant,” substantially enhancing its real-world clinical usability [152].

Except for the prognostic immune risk score, which showed a slightly lower AUC (0.67) in the validation cohort [172], all other models achieved AUC values above 0.7 in both cohorts. Notably, the PISIR model demonstrated exceptionally high predictive accuracy (AUC > 0.9) [152]. However, these models generally suffer from limited sample sizes: only the BICS model included 120 patients [171], while the others enrolled fewer than 100. Although the PISIR model exhibited outstanding predictive performance, its total sample size of only 39 patients indicates that its applicability and generalizability in larger populations remain to be further validated [152].

### Models based on cytokines

Since the predictive power of individual cytokines is relatively limited and remains largely exploratory, some studies have shifted toward developing multi-parameter predictive models that integrate multiple cytokines, achieving notably improved performance. Wang et al. developed a machine-learning model to identify prognostic cytokine signatures associated with nivolumab clearance. Using a training cohort of 468 patients from CheckMate 066 and CheckMate 067 and a validation cohort of 158 patients from CheckMate 037, the model demonstrated that melanoma patients with lower predicted clearance based on selected cytokine features experienced significantly longer survival [168]. Subsequently, Wei et al. constructed the preCIRI14 and edtCIRI19 models in NSCLC patients receiving aPD-1/aPD-L1 monotherapy or combination therapy, based on 14 baseline cytokines and 19 post-treatment cytokines, respectively [174]. Both models consistently showed that patients in the high-risk group had significantly shorter OS than those in the low-risk group. In addition, Gao et al. quantified 92 plasma proteins in 91 patients with esophageal cancer and identified three angiogenesis-related factors (IL-8, angiopoietin-1 receptor and hepatocyte growth factor) to build an angiogenesis-associated risk score model, revealing that high risk score was significantly associated with shorter OS, shorter PFS, and non-response [180].

Evaluation of these cytokine-based models shows that, except for preCIRI14, which had a slightly lower 2-year AUC of 0.568 in the validation cohort, all other models achieved AUCs above 0.7. Notably, edtCIRI19 reached an AUC of 0.9 at the most recent time point and maintained an AUC greater than 0.7 even at two years, demonstrating excellent durability and stability of prediction [174]. Nevertheless, the number of cytokine-based predictive models remains limited, largely due to technical constraints—particularly their reliance on specialized, standardized multiplex protein-detection platforms that are not yet widely adopted in routine clinical practice.

### Integrated models based on multi-omics

With the advancement of technology, the most cutting-edge predictive models are increasingly integrating liquid biopsy data, immune cell profiles, and clinical parameters to achieve higher accuracy in forecasting immunotherapy outcomes.

By integrating baseline plasma bTMB, peripheral CD8^+^ T-cell levels, and early on-treatment ΔctDNA, the DIREct-On model successfully identified a high-score subgroup of NSCLC patients, treated with aPD-1/PD-L1 monotherapy, chemo-immunotherapy, or aPD-1 plus aCTLA-4, who experienced significantly longer PFS [167]. Similarly, Ai et al. constructed a multimodal prediction model incorporating baseline normalized bTMB, early ctDNA dynamics (ΔctDNA), and the first RECIST response, and demonstrated in NSCLC patients treated with various aPD-1/aPD-L1-based regimens that those with higher model scores had significantly longer PFS [181]. In addition, although the LORIS model is built from tissue TMB together with routine clinical and laboratory variables (history of systemic treatment, blood albumin, blood NLR, age and cancer type), it showed robust performance in both the 964-patient training cohort and 515-patient validation cohort, revealing that patients with low LORIS scores (cutoff 0.5) experienced significantly worse OS and PFS [177].

The comparison of model performance shows that the DIREct-On model achieves an AUC of 0.93 [167], markedly outperforming immune-cell-only models except for PISIR [152]. Similarly, the multimodal predictive model developed by Ai et al. demonstrates robust performance, with AUCs of 0.878 and 0.887, reflecting excellent discriminative and predictive capability [181]. Collectively, these studies highlight that integrating tumor-associated and immune-related biomarkers provides a more comprehensive reflection of a patient’s antitumor immune landscape, thereby enhancing predictive precision. However, the clinical translation of such models remains limited. This is primarily due to the high cost and technical complexity of high-throughput sequencing required for ctDNA and bTMB assessment, as well as the lack of prospective validation confirming their independent predictive value.

### Models based on other types of parameters

In addition to tumor- and immune cell-related parameters, several studies have explored novel biomarkers as input variables for multiparametric predictive models. Hurkmans et al. developed a prediction model using partial least squares discriminant analysis (PLS-DA) for melanoma and NSCLC patients treated with nivolumab or pembrolizumab, which effectively distinguished responders from non-responders to immunotherapy by profiling phosphorylation signaling activity in baseline PBMC lysates [169]. In addition, Chen et al. discovered and validated a 5-gene signature model (comprising UQCRB, NDUFA3, CDKN2D, FMNL1-DT, and APOL3) in two independent cohorts. This gene panel demonstrated high predictive accuracy for response to aPD-1 combined with chemotherapy in NSCLC patients (AUC = 0.89) and showed a significant independent association with longer PFS [179]. Collectively, these studies illustrate an innovative expansion of peripheral blood biomarker research toward functional proteomics and transcriptomics, offering a novel mechanistic framework for uncovering predictive biomarkers and refining immunotherapy response models.

Taken together, although these models generally demonstrate good discriminative performance based on their reported metrics, they merely remain at the level of predictive accuracy and have not yet demonstrated their clinical efficacy. Their clinical translatability remains limited by several factors, including insufficient external validation, relatively small sample sizes, and substantial heterogeneity in assay platforms. Considering the current maturity of existing models, together with cost-effectiveness and practical feasibility, the models most likely to be piloted in clinical settings are those based on routine clinical and laboratory parameters, exemplified by the SCORPIO model [182]. However, these models still require validation through prospective randomized controlled trials to establish their clinical utility. In addition, key challenges remain, including variability in data availability, quality, and standardization across institutions, as well as how to effectively integrate such models into routine clinical workflows.

## Conclusion

**Fig. 2:**
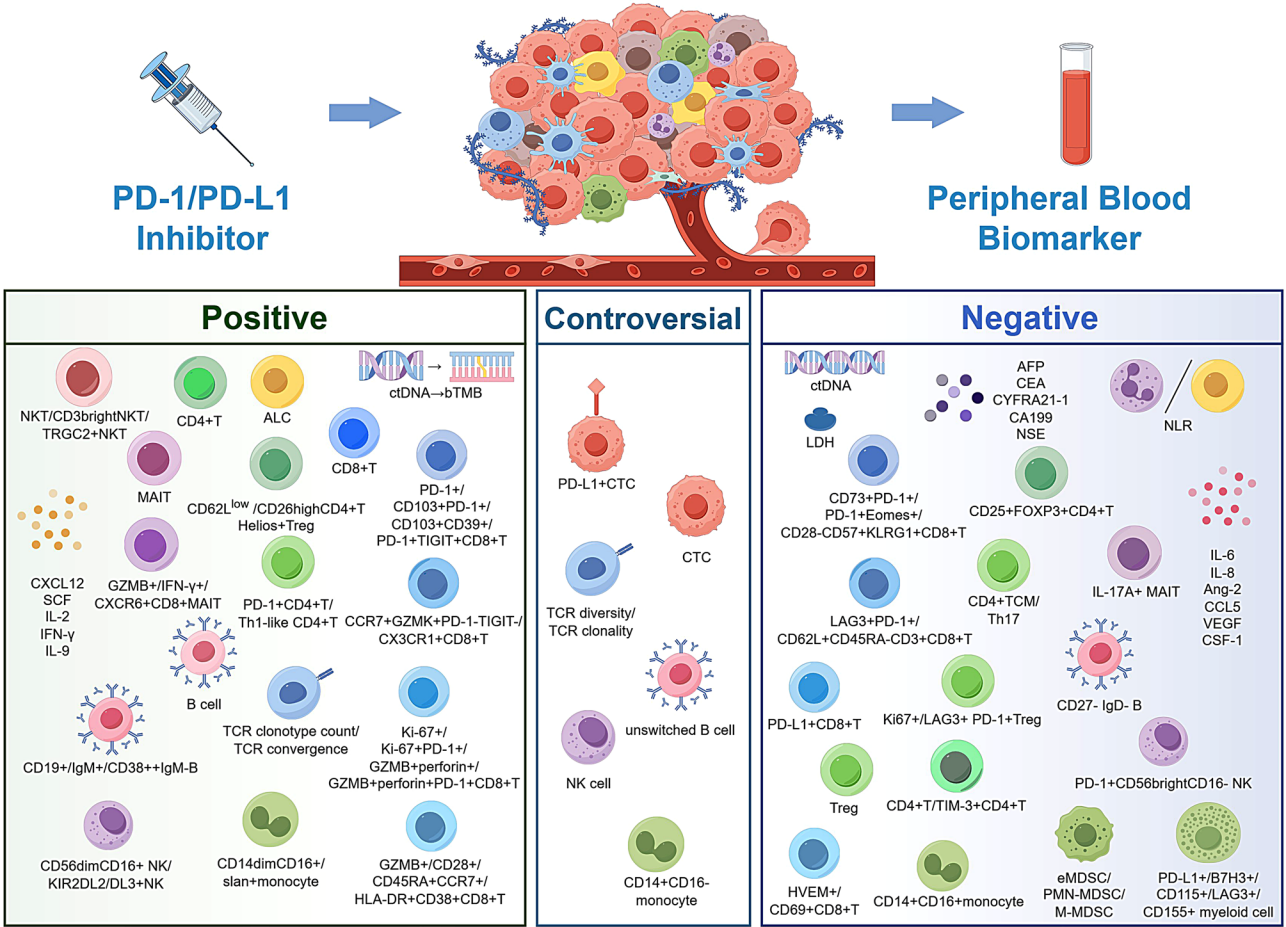
Overview of the peripheral blood biomarkers. The peripheral blood biomarkers identified in this review for predicting response to PD-1/PD-L1 inhibitors can be classified into three types: positive, negative, and controversial

Today, with the rapid development of immunotherapy, biomarkers in peripheral blood can provide supplementary information to existing tumor markers, thereby maximizing the benefits for patients. Here, we summarize some of the latest research findings on peripheral blood as predictive markers for the efficacy of aPD-1/aPD-L1 treatments (Fig. 2).

Based on the evidence levels extracted from the included studies, no mature Level-1 biomarker currently exists to support regulatory approval or inclusion in clinical guidelines. Nonetheless, several indicators are progressing toward higher-level validation. Tumor-related markers such as CEA and CYFRA21-1 [23], CTCs [39, 40] and PD-L1^+^ CTCs [43], together with selected immune subsets including CD73^+^ PD-1^+^ CD8^+^ T cells [89] and CD26highCD4 +T cells [110], have been prospectively evaluated and reached level-2A evidence. Similarly, ctDNA [26, 30, 31] and bTMB [34–37] have demonstrated consistent associations in exploratory analyses of large randomized trials, while CD62L^low^ CD4+T cells and CD25^+^ FOXP3^+^ CD4^+^ T cells [109] showed strong correlations with outcomes in large prospective cohorts, forming level-2B evidence. These Level-2 biomarkers have generated robust biological and clinical hypotheses and now require confirmation in randomized trials with standardized assays and validated cut-offs to facilitate clinical translation.

Level-3 retrospective evidence mainly involves clinically accessible and cost-effective indices such as tumor markers, LDH, lymphocyte counts, and NLR. Despite their practicality, their predictive value remains limited by non-specificity and should be validated in prospective, multi-center cohorts, ideally in combination with molecular or immune markers to improve specificity and robustness. Level-4 biomarkers, including emerging immune phenotypes, TCR clonality, and cytokine signatures, remain in early discovery stages and are still distant from clinical application.

For PD-1/PD-L1 Inhibitors monotherapy, key predictive biomarkers encompass both tumor burden—mainly reflected by tumor markers [23, 24], CTCs [38–40], PD-L1^+^ CTCs [43, 44], and ctDNA [26, 31]—and immune cell subsets that exhibit either cytotoxic potential, such as CD103+PD-1+CD8+T cells [86], GZMB+MAIT and IFN-γ+MAIT cells [123], NKT cells [125, 126], and CD56^dim^CD16+NK [148, 149] cells, or immunosuppressive/exhausted phenotypes, including CD73+PD-1+CD8+T cells [89], PD-1+Eomes+CD8+T cells [90], Tregs [109], IL-17A+ MAIT cells [123], and inhibitory myeloid populations [154]. Together, these markers provide an integrated profile of tumor-mediated immune suppression and the potential of ICIs to reinstate anti-tumor effector responses.

Biomarkers for combination therapy also focus on tumor-related factors and anti-tumor immunity, yet are distinct from those for monotherapy. In PD-1/PD-L1 Inhibitors–chemotherapy combinations, dynamic ctDNA changes emerge as sensitive indicators [32, 33], while phenotypes linked to trafficking (CCR7+GZMK+PD-1-TIGIT-CD8+T cells [92], CD4+CCR4+CCR6+T cells [95], CXCR6+CD8+MAIT cells [124]) or early memory differentiation (CD8+/CD4+ TCM [95, 116]) align with chemotherapy-induced antigen release and enhanced immune priming [107]. The representative markers of ICI–targeted therapy are proliferative effector cells (Ki-67+CD8+ T and Ki-67+PD-1+CD8+ T [97, 100]) and persistent suppressive Treg subsets (LAG3^+^ PD-1^+^ Tregs [97]), consistent with their role in remodeling the immunosuppressive microenvironment. Across all three treatment types, NLR and IL-6 demonstrate stable predictive value. Evidence for chemoradiotherapy plus ICIs is scarce; existing studies highlight suppressive cells (Tregs, MDSCs [117, 118]) and inhibitory cytokines (IL-6, IL-8 [117, 159]), reflecting the balance between ICI-driven activation and chemoradiotherapy-induced immunosuppression. Double-ICI combinations [29, 35, 157] and multi-modality regimens [21, 28, 41] remain limited, with ctDNA, bTMB, IL-6 and AFP, CTC, ctDNA emerging as predictors, underscoring the broad applicability of tumor-related markers.

With the rapid advancement of artificial intelligence and algorithmic modeling, along with the growing availability of clinical and molecular data, multi-parameter models have emerged as an inevitable direction in the development of peripheral blood biomarkers. Current evidence suggests that integrating robust single biomarkers—such as ctDNA or bTMB—into models based on immune-cell features or clinical characteristics may improve predictive performance, highlighting the role of such biomarkers as foundational components in constructing more comprehensive models. However, it is important to note that multi-parameter models are still in the early stages of exploration, having started later than single-parameter approaches. Moreover, as no single peripheral blood biomarker has yet entered routine clinical use or large-scale randomized trials, existing studies are marked by heterogeneity in parameter selection, model design, and patient cohorts. Consequently, multi-parameter models currently lack the conditions for widespread application in research and are not yet suitable for comparing differences between PD-1/PD-L1 inhibitor monotherapy and combination therapy. There remains a considerable journey ahead before these models can be effectively implemented in clinical practice.

Based on the above conclusion, in the short term, research should prioritize biomarkers with strong biological plausibility but pending clinical validation, such as ctDNA and bTMB, focusing on randomized trials, assay standardization, platform harmonization, and the establishment of clinically meaningful cut-offs. Clinically promising multiparameter models (e.g., the SCORPIO model) should be prospectively tested in real-world settings to evaluate generalizability. In the long term, research should move toward developing dynamic multi-omics integration frameworks. By incorporating genomic, immunomic, cellular, and clinical data, these systems can generate continuously updated multi-parameter models to more precisely dissect tumor–immune interactions. In addition, establishing cross-platform and multi-center data-sharing infrastructures will be essential to accelerate biomarker validation and clinical translation.

## Data Availability

No datasets were generated or analysed during the current study.

## Abbreviations

ACC: adrenal cortical carcinoma
AFP: Alpha-fetoProtein
ALC: absolute lymphocyte count
aPD-1: anti-PD-1
aPD-L1: anti-PD-L1
AUC: area under curve
AVD: doxorubicin, vinblastine and dacarbazine
BOR: best overall response
BSC: best supportive care
bTMB: blood tumor mutation burden
CA199: carbohydrate antigen 19-9
CBR: clinical benefit rate
CCR: correct classification rate
ccRCC: clear cell renal cell carcinoma
CD4+TCM: central memory CD4+ T cell
CEA: carcinoembryonic antigen
CHL: classic Hodgkin lymphoma
C-index: concordance index
CPS: combined positive score
CR: complete response
CRC: colorectal cancer
CRT: chemoradiation therapy
CTC: circulating tumor cell
ctDNA: circulating tumor DNA
CYFRA21-1: cytokeratin 19 fragment antigen21 -1
DCR: disease control rate
DFS: disease-free survival
EAC: esophageal adenocarcinoma
ECOG PS: Eastern Cooperative Oncology Group Performance Status
eMDSC: early myeloid-derived suppressor cell
ESCC: esophageal squamous cell carcinoma
FDA: Food and Drug Administration
FOLFOX: folinic acid, fluorouracil and oxaliplatin
G/GEJ: adenocarcinoma gastric and gastroesophageal junction adenocarcinoma
GAC: gastric adenocarcinoma
GC: gastric cancer
HCC: hepatocellular carcinoma
HER2+ EGA: HER2-positive esophagogastric adenocarcinoma
hGE: haploid genome equivalent
HNSCC: head and neck squamous cell carcinoma
ICI: Immune checkpoint inhibitor
LDH: lactate dehydrogenase
MAIT: mucosal-associated invariant T cell
mCRC: metastatic colorectal cancer
MDSC: myeloid-derived suppressor cell
MFS: metastasis-free survival
MHC: major histocompatibility complex
MIBC: muscle-invasive bladder cancer
M-MDSC: monocytic myeloid-derived suppressor cell
MPLC: multiple primary lung cancer
MPR: major pathological response
MSI-H: microsatellite instability-high
mVAF: median variant allelic frequency
neo: neoadjuvant
NEPC: neuroendocrine prostate cancer
NK: natural killer cell
NKT: natural killer T cell
NLR: neutrophil-to-lymphocyte ratio
NPC: nasopharyngeal carcinoma
NSE: neuron-specific enolase
ORR: objective response rate
OS: overall survival
PAAD: pancreatic adenocarcinoma
PBMC: peripheral blood mononuclear cell
pCR: pathological complete response
PD: progressive disease
PD-1: programmed death 1
PD-L1: programmed cell death ligand 1
PD-L1+CTC: PD-L1-positive circulating tumor cells
PFS: progression-free survival
PMN-MDSC: polymorphonuclear myeloid-derived suppressor cell
PR: partial response
RCC: renal cell carcinoma
RCT: randomized controlled trial
RECIST: response evaluation criteria in solid tumors
SBRT: stereotactic body radiation therapy
SCBC: small cell bladder cancer
SCLC: small cell lung cancer
SD: stable disease
SII: systemic immune inflammation index
SoC: cetuximab, a taxane, methotrexate, or a fluoropyrimidine
SOX: S-1 and oxaliplatin
STS: soft tissue sarcomas
TACE: transarterial chemoembolization
TCR: T cell receptor
TKI: tyrosine kinase inhibitor
TMB: tumor mutation burden
Treg: regulatory T cell
UC: urothelial carcinoma
ULN: upper limit of normal
